# Strain displacement in microbiomes via ecological competition

**DOI:** 10.1038/s41564-025-02162-w

**Published:** 2025-11-07

**Authors:** Erik Bakkeren, Vit Piskovsky, Megan N. Y. Lee, Martin T. Jahn, Kevin R. Foster

**Affiliations:** 1 Department of Biology, University of Oxford, Oxford, UK; 2 Department of Biochemistry, University of Oxford, Oxford, UK; 3 Sir William Dunn School of Pathology, University of Oxford, Oxford, UK; 4 Department of Mathematics, University of Oxford, Oxford, UK; 5 Present Address: Department of Biological Sciences, University of Calgary, Calgary, Alberta Canada; 6 Present Address: Department of Environmental Microbiology, Eawag, Dubendorf, Switzerland; 7 Present Address: Institute of Biogeochemistry and Pollutant Dynamics, Department of Environmental Systems Science, ETH Zurich, Zurich, Switzerland; 8 Present Address: Bacterial Infection Ecology, Helmholtz Center for Infection Research, Braunschweig, Germany

**Keywords:** Microbiome, Microbial ecology

## Abstract

Microorganisms commonly live in diverse communities where changes in composition can be critical for health, industry and the environment. Yet, what enables one strain to competitively replace another in these complex conditions remains poorly understood. Here we develop a mathematical model to determine general principles of strain displacement. Our modelling reveals that weak resource competition enables successful invasion while strong interference competition, for example, via antimicrobial production, enables successful displacement. We verify these predictions using in vitro assays with genetically engineered *Escherichia coli*. We then apply our principles to displace multidrug-resistant clinical isolates using strains that are equipped with a potent bacteriocin. Finally, we perform experiments with diverse human gut symbionts, which reveal that displacement relies on low resource competition not only between competing strains but also with the broader community, that is, limited nutrient blocking. These general rules for ecological success in microbial communities could be applied for targeted displacement of bacteria.

## Abstract (web summary)

Mathematical modelling and experimental tests reveal principles that govern displacement of a resident strain by an invader in microbial communities.

## Main

Bacterial communities, or microbiomes, occur almost everywhere and are important for many aspects of our lives, including industrial processes, the environment and the balance between health and disease^1–3^. A key determinant of the impacts of microbial communities is their composition; changes in the identity of strains and species can rapidly shift a beneficial community to a harmful one—for example, displacement of protective gut bacteria by pathogenic bacteria, or vice versa^4–7^. A key goal for the field, therefore, is to understand what makes strains or species successful within an established microbiome. To investigate this, we ask what it takes for an incoming strain to invade and displace a competing strain that is established in a community.

A key challenge with understanding microbial communities is their complexity. Diverse strains and species commonly co-occur and interact ecologically, which can generate context dependencies that hinder generalization^3,8,9^. What is clear is that ecological competition, particularly between strains that have overlapping resource requirements, is a key process shaping microbial communities^10^. Most fundamentally, nutrient competition is considered important for assembly and stability in systems such as the human microbiome^11–14^ as well as resistance to invasion by pathogens^15–17^. In addition, many bacterial species engage in interference competition via a diverse range of dedicated competition systems, or ‘weapons’, that typically function by delivering a toxin to competing strains^18^. Evidence that these weapons kill is widespread^18,19^ along with evidence that they shape ecological dynamics within communities^4,6,20^. Weapon-bearing bacteria are also attractive as biotherapeutics^21,22^ and alternatives to conventional antibiotics. A key advantage is that many bacterial toxins are narrow spectrum and can target a harmful strain while leaving the broader community largely unaffected^23^. However, while both nutrient competition and interference competition can be individually important, how they act in concert is not well understood.

Here we build a general ecological model to understand strain competition in a microbial community. Our modelling framework identifies specific ecological conditions that enable a new strain to establish in a community and out-compete a resident strain, which can lead to strain turnover and displacement. We test our modelling predictions with the gut bacterium *Escherichia coli*, which is both a model species and a dominant cause of mortality from antimicrobial-resistant (AMR) infections^24^. We perform these tests with *E. coli* strains alone and in a diverse community of human gut bacteria. In this way, we are able to establish general principles underlying competitive strain displacement within microbial communities.

## Results

### Ecological theory predicts conditions for strain invasion and displacement

We are interested in what enables a microbial strain to invade an established community and out-compete an existing strain. Mathematical models, particularly from theoretical ecology, have proved to be a powerful way to cut through the complexity of microbial communities and identify general predictions^10,14,23,25–27^. We begin, therefore, by building a general mathematical framework based upon previous theoretical work of resource competition in microorganisms and plants^23,28,29^ (Supplementary Information). Following the biology of microbial communities such as the human gut microbiome, the models capture two key forms of ecological competition. Firstly, we assume that the newly arriving strain will experience competition for resources^30,31^, where its growth rate upon arrival depends upon the level of the nutrients needed for growth^15,17,32,33^. Secondly, we include the possibility that our focal strain may engage in interference competition, which can be either via the release of toxins into the environment or via a contact-dependent mechanism such as the type six secretion system^18,34^.

**Fig. 1:**
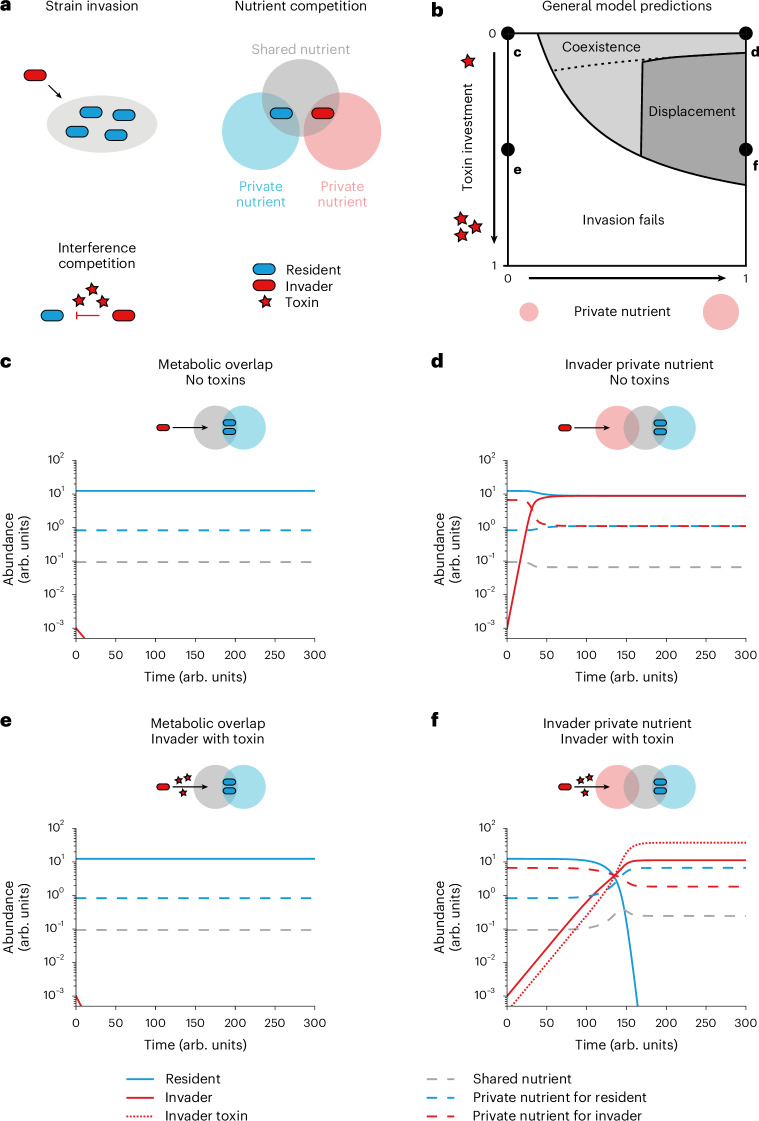
Ecological theory predicts conditions for strain invasion and displacement. **a**, Our theory captures three key aspects of the natural ecology of bacterial competition: ecological strain invasions in which an invader (red) invades the niche of a resident (blue), nutrient competition over a shared nutrient (grey) and interference competition (red stars for toxins produced by the invader). **b**, Invasion success is the result of varying toxin investment (*z*) or the supplementation of a private nutrient for an invader (*m*_I_). Invasion fails (white region) if private nutrients are not sufficiently available. If an invader has sufficient access to a private nutrient, it can coexist with a resident strain (light grey region), but if it invests sufficiently into a toxin, it can displace the resident strain (dark grey region). The invasion boundary is analytically determined (Supplementary Equation (12), Supplementary Text), and the displacement boundary is plotted numerically (Methods), with the dashed line delimiting the analytically derived bound for the displacement boundary (Supplementary Equation (17), Supplementary Text). **c**–**f**, Numerical solutions of points in **b** in which the invader has not invested in the production of a toxin and has no private nutrient (**c**; *m*_I_ = 0, *z* = 0), has not invested in the production of a toxin but has an abundant private nutrient (**d**; *m*_I_ = 1, *z* = 0), has invested in a toxin but has no private nutrient (**e**; *m*_I_ = 0, *z* = 0.5) and has both invested in a toxin and has an abundant private nutrient (**f**; *m*_I_ = 1, *z* = 0.5). Solid lines indicate the abundance of strains (resident in blue, invader in red), the red dotted line indicates the abundance of the invader toxin and the dashed lines indicate the abundance of nutrients (shared nutrient in grey, private nutrient for the resident in blue, private nutrient for the invader in red). Parameter values used for simulating the invasion dynamics from equation (3) (Methods): $$m={m}_{{\rm{R}}}=1,\delta =D=d=0.15,\,{R}_{{\rm{R}}}=$$$${r}_{{\rm{R}}}={R}_{{\rm{I}}}={r}_{{\rm{I}}}=1,{C}_{{\rm{R}}}={c}_{{\rm{R}}}={C}_{{\rm{I}}}={c}_{{\rm{I}}}=1,\,s=1,\,$$$${k}_{{\rm{R}}}={k}_{{\rm{I}}}=1,\,{K}_{{\rm{R}}}={K}_{{\rm{I}}}=K=10,\,g=1,\,p=0.7$$.

Although the impacts of nutrient and interference competition on microbial ecology have been widely studied^11,14,17,34–38^, how they act in combination has not. By combining all three key elements in our modelling—ecological invasion, nutrient competition and interference competition—we can investigate what is required for a strain to invade and out-compete a resident strain in a microbial community (Fig. 1a). We first consider a general consumer-resource model

1 $$\begin{array}{c}\frac{d{N}_{\sigma }}{dt}={N}_{\sigma }({\lambda }_{\sigma }({\bf{N}},{\bf{x}})-{\delta }_{\sigma })\\ \frac{d{x}_{i}}{dt}={g}_{i}({\bf{N}},{\bf{x}})-{\sum }_{\sigma }{N}_{\sigma }{d}_{i\sigma }({\bf{N}},{\bf{x}}),\end{array}$$

where *N*_*σ*_ is the abundance of strain *σ*; *x*_*i*_ is the abundance of nutrient or toxin *i*; *λ*_*σ*_(**N**, **x**) is the per capita growth rate of strain *σ*; *δ*_*σ*_ is the dilution rate of strain *σ*; *g*_*i*_(**N**, **x**) is the net nutrient influx rate or toxin production rate, meaning the rate at which chemical species are introduced into the system; and *d*_*i*__*σ*_(**N**, **x**) is the uptake rate of nutrient or toxin *i* by strain *σ*. This general consumer-resource model captures nutrient competition by a positive dependence of growth rate *λ*_*σ*_ on limited, shared nutrients *x*_*i*_, while interference competition is captured by a negative dependence of growth rate *λ*_*σ*_ on diffusible toxins *x*_*j*_ or competitors *N*_*σ*__′_ that carry contact-dependent weapons. The model reveals important differences between the two forms of ecological competition when a strain invades. Specifically, we show (Theorem 1, Supplementary Information) that a rare invading strain *τ* can invade a community of strains with equilibrium abundances *N*_1_, …, *N*_*τ*−1_ and nutrient or toxin abundances *x*_1_, …, *x*_*I*_ precisely when its initial growth rate overcomes its dilution, that is

2 $${\lambda }_{\tau }\left({N}_{1},\ldots ,{N}_{\tau -1},{N}_{\tau }=0,\,{x}_{1},\ldots ,{x}_{I}\right) > {\delta }_{\tau }.$$

Therefore, as weapons of the invading strain do not directly increase its own initial growth rate *λ*_*τ*_ but rather decrease the growth rates *λ*_*σ*_ of other susceptible strains *σ*, a rare invader strain that is equipped with a weapon cannot invade the community unless it can invade without it (Theorem 1, Supplementary Information). Put another way, the conditions for invasion are independent of whether the focal strain is using interference competition—whether it is a contact-dependent system or diffusing an antimicrobial toxin. This result is true for both well-mixed conditions (Theorem 1) and spatially structured environments (Theorem 2, Supplementary Information). The prediction arises because the benefits of interference competition scale with population size, such that when a strain is sufficiently rare, their impacts on other strains are vanishingly small, and instead, the user merely suffers from the cost associated with production of bacterial weapons. Instead, the model predicts that invasion is determined by the ability to compete for nutrients sufficiently well to achieve a sufficient initial growth rate *λ*_*τ*_. In the model, this condition occurs when there is enough of a nutrient *x*_*i*_ that the invading strain can use but that the resident microorganisms cannot, which we refer to here as a ‘private’ nutrient. Finally, if a strain is invading a community where resident strains are producing antimicrobial toxins, its growth rate also will have to be high enough to overcome any mortality cost from the antimicrobial. This result means that we can approximate the effects of a resident’s toxin using the growth rate of the invader at invasion, and we therefore do not explicitly study the effects of residents’ toxins further.

**Extended Data Fig. 1:**
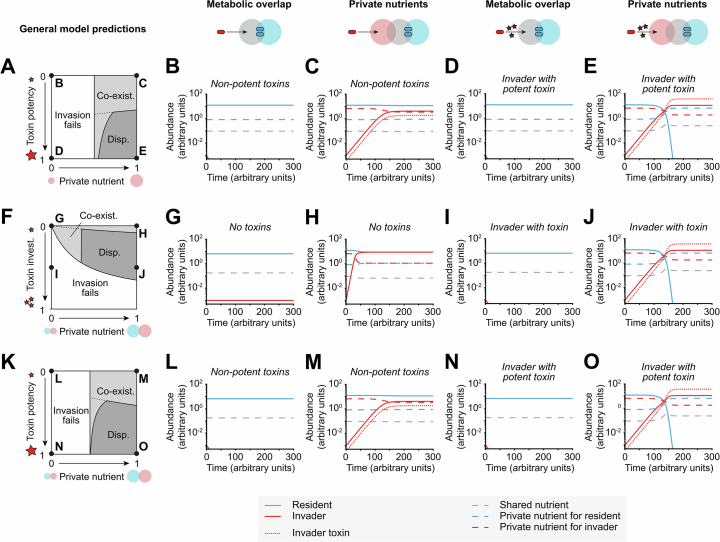
Ecological theory of strain replacement varying toxin potency instead of toxin investment, and varying supplementation of private nutrients for both strains instead of just the invader. Additional scenarios related to Fig. 1 that capture three key aspects of the natural ecology of bacterial competition: ecological strain invasions where an invadNumerical solutions of four scenarioser (red) invades the niche of a resident (blue), nutrient competition over shared nutrient (grey), and interference competition (red stars for toxins produced by the invader). A-E) Varying toxin potency instead of toxin investment. **F-J)** Varying toxin investment, but now varying supplementation of private nutrients for both strains instead of just the invader. **K**-**O**) Varying toxin potency as well as varying supplementation of private nutrients for both strains. **A**,**F**,**K**) Invasion success as a result of varying toxin investment (*z*; abbreviation=invest.) or potency (*p*) on the y-axis, or the supplementation of a private nutrient for the invader (*m*_*I*_) or both the invader and the resident (*m*_*I*_*=m*_*R*_) on the x-axis. Invasion fails (white region) if private nutrients are not sufficiently available. If an invader has sufficient access to a private nutrient, it can co-exist with a resident strain (light grey region), but if it invests sufficiently into a toxin or has a sufficiently potent toxin, it can displace the resident strain (dark grey region; abbreviation=disp.). The invasion boundary is analytically determined (Eq. S12; see Supplementary text) and the displacement boundary is plotted numerically (see Methods), with the dashed line delimiting the analytically derived bound for the displacement boundary (Eq. S17; see Supplementary text). **B**-**E**,**G**-**J**,**L**-**O**) Numerical solutions of four scenarios panels **A**,**F**,**K**, indicated as black points. Solid lines indicate the abundance of strains (resident in blue, invader in red), the dotted red line indicates the abundance of the invader toxin, and the dashed lines indicate the abundance of nutrients (shared nutrient in grey, private nutrient for the resident in blue, private nutrient for the invader in red). **B-E)** The invader has a non-potent toxin and no private nutrient (**panel B**; *m*_*I*_=0, *p*=0), a non-potent toxin but an abundant private nutrient (**panel C**; *m*_*I*_=1, *p*=0), a potent toxin but no private nutrient (**panel D**; *m*_*I*_=0, *p*=1), and a potent toxin and an abundant private nutrient (**panel E**; *m*_*I*_=1, *p*=1). Parameter values used for simulating the invasion dynamics from Eq. 3 (**Methods**): $$m={m}_{R}=1,\delta =D=d=0.15,{R}_{R}={r}_{R}={R}_{I}=$$$${r}_{I}=1,{C}_{R}={c}_{R}={C}_{I}={c}_{I}=1,s=1,{k}_{R}={k}_{I}=$$$$1,{K}_{R}={K}_{I}=K=10,z=0.5,g=1.$$ **G-J)** The invader has not invested in the production of a toxin and there are no private nutrients (**panel G**; *m*_*I*_*=m*_*R*_ =0, *z*=0), the invader has not invested in the production of a toxin but there are private nutrients for both strains (**panel H**; *m*_*I*_*=m*_*R*_ =1, *z*=0), the invader invested in a toxin but there are no private nutrients (**panel I**; *m*_*I*_*=m*_*R*_=0, *z*=0.5), and the invader has both invested in a toxin and there are private nutrients (**panel J**; *m*_*I*_*=m*_*R*_ =1, *z*=0.5). Parameter values used for simulating the invasion dynamics from Eq. 3**(Methods)**: $$m=1,\delta =D=d=0.15,{R}_{R}={r}_{R}={R}_{I}={r}_{I}=$$$$1,{C}_{R}={c}_{R}={C}_{I}={c}_{I}=1,s=1,{k}_{R}={k}_{I}=$$$$1,{K}_{R}={K}_{I}=K=10,g=1,p=0.7.$$ **L-O)** The invader does not have a potent toxin and there are no private nutrients for either strain (**panel L**; *m*_*I*_*=m*_*R*_ =0, *p*=0), the invader does not have a potent toxin but there are private nutrients for both strains (**panel M**; *m*_*I*_*=m*_*R*_ =1, *p*=0), the invader has a potent toxin but there are no private nutrients for either strain (**panel N**; *m*_*I*_*=m*_*R*_ =0, *p*=1), the invader has a potent toxin and there are private nutrients for both strains (**panel O**; *m*_*I*_*=m*_*R*_ =1, *p*=0). Parameter values used for simulating the invasion dynamics from Eq. 3**(Methods)**: $$m=1,\delta =D=d=0.15,{R}_{R}={r}_{R}={R}_{I}={r}_{I}=$$$$1,{C}_{R}={c}_{R}={C}_{I}={c}_{I}=1,s=1,{k}_{R}={k}_{I}=$$$$1,{K}_{R}={K}_{I}=K=10,z=0.5,g=1$$.

**Extended Data Fig. 2:**
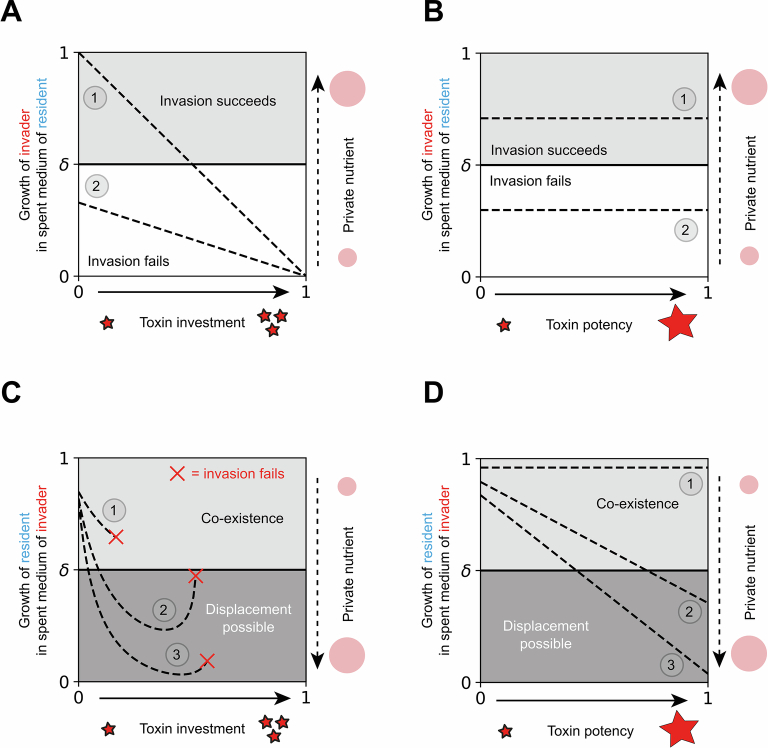
Invasibility conditions. Invader strain denoted by red and resident strain denoted by blue. **A)** Impact of toxin investment *z* on invasion success. Invasion success decreases with investment into toxin production (dashed lines) and increases with the supplementation of private nutrients to the invader. Dashed lines correspond to the growth rate of the invader in the spent medium of the resident (Eq. S12) and $$\delta =0.5$$ denotes the dilution rate ($$p=0.7$$, (1): $${m}_{I}=0.1$$, (2): $${m}_{I}=1.5$$). **B)** Impact of toxin potency*p* on invasion success. Invasion success is independent of toxin potency and increases with the supplementation of private nutrients to the invader. Dashed lines correspond to the growth rate of the invader in the spent medium of the resident (Eq. S12) and $$\delta =0.5$$ denotes the dilution rate ($$z=0.5$$, (1): $${m}_{I}=0.4$$, (2): $${m}_{I}=4$$). **C)** Impact of toxin investment *z* on displacement. The possibility of displacement increases with the supplementation of private nutrients and a suitable toxin investment, as long as the metabolic cost of toxin production does not prevent the success of invasion (red cross). Dashed lines correspond to the growth rate of the resident in the spent medium of the invader (Eq. S17) and $$\delta =0.5$$ denotes the dilution rate ($$p=0.7$$, (1): $${m}_{I}=0.5$$, (2): $${m}_{I}=2$$, (3): $${m}_{I}=4$$). **D)** Impact of toxin potency *p* on displacement. The possibility of displacement increases with toxin potency and supplementation of private nutrients. Dashed lines correspond to the growth rate of the resident in the spent medium of the invader (Eq. S17) and $$\delta =0.5$$ denotes the dilution rate ($$z=0.5$$, (1): $${m}_{I}=1.5$$, (2): $${m}_{I}=2$$, (3): $${m}_{I}=4$$). Parameter values used for plotting this figure: $$m={m}_{R}=1,\delta =0.5,D=d=0.15,{R}_{R}=$$$${R}_{I}=2,{r}_{R}={r}_{I}=1,{C}_{R}={c}_{R}={C}_{I}=$$$${c}_{I}=1,s=1,{k}_{R}={k}_{I}=1,{K}_{R}={K}_{I}=K=$$$$10,z=0.5,g=1,p=0.7$$.

**Extended Data Fig. 3:**
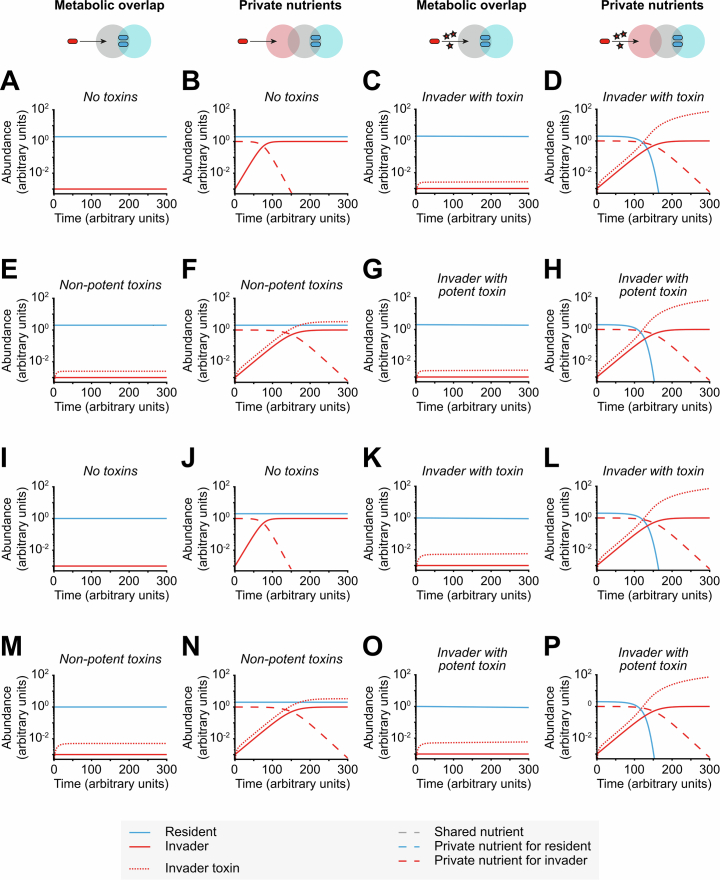
Numerical simulations of the batch culture dynamics. Multiple numerical solutions of batch culture dynamics are presented where the variable toxin investment and variable availability of private nutrient (**panels A-D**), variable toxin potency and variable availability of private nutrient (**panels E-H**), variable toxin investment and both invader and resident have variable availability of private nutrients (**panels I-L**), and variable toxin potency and both invader and resident have variable availability of private nutrients (**panels M-P**). Toxin investment is varied between values $$z=0$$ ((**panels A, B, I, J**) and $$z=0.5$$ (**panels C, D, K, L**), while toxin potency is varied between values $$p=0$$ (**panels E, F, M, N**) and $$p=1$$ ((**panels G, H, O, P**). Similarly, the availability of the invader’s private nutrient is varied between initial values *m*_*I*_ = 0 (**panels A, C, E, G**) and *m*_*I*_ = 1 (**panels B, D, F, H**), while the private nutrients of both invader and resident are varied between initial values *m*_*I*_ = *m*_*R*_ = 0 (**panels I, K, M, O**) and *m*_*I*_ = *m*_*R*_ = 1 (**panels J, L, N, P**). Moreover, the initial abundance of the shared nutrient is always set to a value *m* = 1. After the resident strain, introduced at initial abundance of *N*_*R*_ = 0.001, reaches an equilibrium, the invader strain is introduced at abundance *N*_*I*_ = 0.001 and the resulting dynamics is plotted. Solid lines indicate the abundance of strains (resident in blue, invader in red), the dotted red line indicates the abundance of the invader toxin, and the dashed lines indicate the abundance of nutrients (shared nutrient in grey, private nutrient for the resident in blue, private nutrient for the invader in red). Baseline parameter values used for simulating the invasion dynamics from Eq. 3**(Methods)**: $$m={m}_{R}={m}_{I}=0,\delta =D=d=0,{R}_{R}=$$$${r}_{R}={R}_{I}={r}_{I}=1,{C}_{R}={c}_{R}={C}_{I}={c}_{I}=$$$$1,s=1,{k}_{R}={k}_{I}=1,{K}_{R}={K}_{I}=$$$$K=10,g=1,p=0.7,z=0.5$$.

Next, to illustrate the general modelling predictions, we numerically simulate the ecological dynamics that occur after a strain invades (Fig. 1c–f and Extended Data Figs. 1 and 2). The main figures (Fig. 1c–f) show the dynamics of the continuous flow version of our model, which is the form that allowed steady-state calculations and the most analytical tractability (equations (1) and (2)). In the supplement (Extended Data Fig. 3), we show that the same predictions hold for a batch culture version of our model, which is the basis of our experimental work (below). For simplicity, our model captures the resident community as a single strain that has an overlapping ecological niche with the invader (Methods).

**Extended Data Fig. 4:**
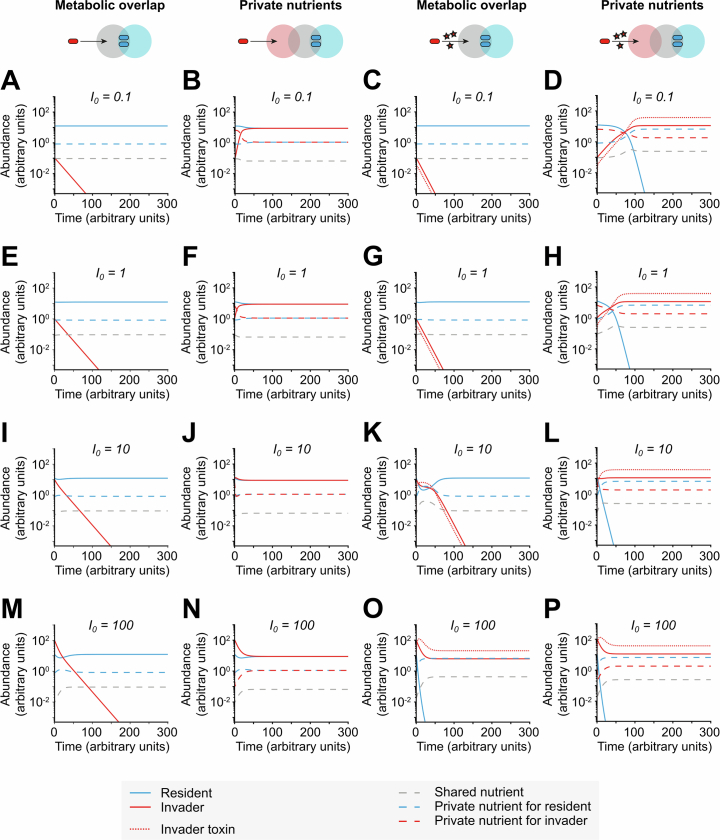
Numerical simulations varying invader starting density. Extension of Fig. 1 to variable initial abundance of invaders. Invasion success is plotted as a result of varying toxin investment (*z*), the supplementation of a private nutrient for an invader (*m*_*I*_) and the initial invader abundance (*I*_*0*_). The first column shows the case where the invader has not invested in the production of a toxin and has no private nutrient (**panels A,E,I,M**; *m*_*I*_=0, *z*=0). The second column shows the case where the invader has not invested in the production of a toxin but has an abundant private nutrient (**panels B,F,J,N**; *m*_*I*_=1, *z*=0). The third column shows the case where the invader has invested in a toxin but has no private nutrient (**panels C,G,K,O**; *m*_*I*_=0, *z*=0.5). The fourth column shows the case where the invader and has both invested in a toxin and has an abundant private nutrient (**panels D,H,L,P**; *m*_*I*_=1, *z*=0.5). Different rows correspond to different initial abundances of invaders (*I*_*0*_=0.1, 1, 10, 100), which are indicated in the title of each panel. Solid lines indicate the abundance of strains (resident in blue, invader in red), the dotted red line indicates the abundance of the invader toxin, and the dashed lines indicate the abundance of nutrients (shared nutrient in grey, private nutrient for the resident in blue, private nutrient for the invader in red). Parameter values used for simulating the invasion dynamics from Eq. 3 (**Methods**): $$m={m}_{R}=1,\delta =D=d=0.15,{R}_{R}=$$$${r}_{R}={R}_{I}={r}_{I}=1,{C}_{R}={c}_{R}={C}_{I}=$$$${c}_{I}=1,s=1,{k}_{R}={k}_{I}=1,{K}_{R}={K}_{I}=$$$$K=10,g=1,p=0.7$$.

**Extended Data Fig. 5:**
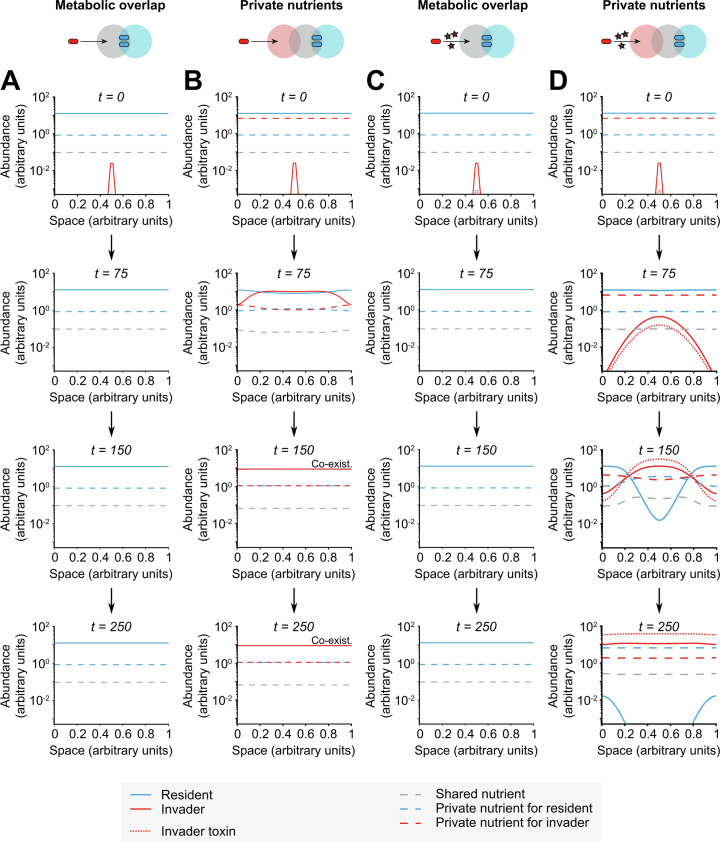
Numerical simulations of the spatially explicit model. Extension of Fig. 1 to spatially explicit contexts. Numerical simulated spatial profiles of Eq. S27 are shown in different rows at specified timepoints (*t*=0, 75, 150, 250). Different panels correspond to the invader that has not invested in the production of a toxin and has no private nutrient (**panel A**; *m*_*I*_=0, *z*=0), has not invested in the production of a toxin but has an abundant private nutrient (**panel B**; *m*_*I*_=1, *z*=0), has invested in a toxin but has no private nutrient (**panel C**; *m*_*I*_=0, *z*=0.5), and has both invested in a toxin and has an abundant private nutrient (**panel D**; *m*_*I*_=1, *z*=0.5). Solid lines indicate the abundance of strains (resident in blue, invader in red), the dotted red line indicates the abundance of the invader toxin, and the dashed lines indicate the abundance of nutrients (shared nutrient in grey, private nutrient for the resident in blue, private nutrient for the invader in red). In **panel B**, the lines corresponding to residents and invaders are on top of each other, which is indicated by the word “co-exist.” Parameter values used for simulating the invasion dynamics from Eq. S27: $$m={m}_{R}=1,\delta =D=d=0.15,{R}_{R}={r}_{R}=$$$${R}_{I}={r}_{I}=1,{C}_{R}={c}_{R}={C}_{I}={c}_{I}=1,s=$$$$1,{k}_{R}={k}_{I}=1,{K}_{R}={K}_{I}=K=10,g=1,$$$$p=0.7,{M}_{NR}={M}_{NI}=0.0001,{M}_{x}=$$$${M}_{xI}={M}_{xR}=0.01,{M}_{y}=0.001$$.

Consistent with our analytics, we see that the invasion of the focal strain depends upon its having a private nutrient, but invasion does not depend upon the use of an antimicrobial toxin (Fig. 1b–d). However, the toxin is critical for the outcome of competition over longer time periods. Specifically, in the absence of a toxin, strain invasion leads to coexistence of the two strains (Fig. 1e). By contrast, when the invader invests sufficiently into toxin production, strain invasion leads to the removal of the resident and strain displacement (Fig. 1b; compare panels d and f of Fig. 1). We further confirm that displacement occurs because of increased density of the invading strain as it grows on its private nutrient by showing that a sufficient initial density of invaders can cause displacement of a resident strain without a private nutrient for the invader (Extended Data Fig. 4). To explore Theorem 2 (Supplementary Information), we also performed spatially explicit simulations in which the resident strain is stable and spatially homogeneous, and the invader is seeded at low density along a spatial axis (Extended Data Fig. 5). As for well-mixed conditions, a rare invading strain can grow only if it has a private nutrient, and it is these conditions that empower it to displace a resident strain if it uses interference competition.

### Strain displacement occurs as predicted with engineered *E. coli* strains

To test our modelling predictions, we turned to *E. coli* as a model organism. This species is both amenable to genetic manipulation and a good test case because competition among strains can be critical for human health: some *E. coli* strains are harmless members of the gut microbiome, while others cause deadly diseases whose treatment is hindered by the rising incidence of AMR^24,39,40^.

**Fig. 2:**
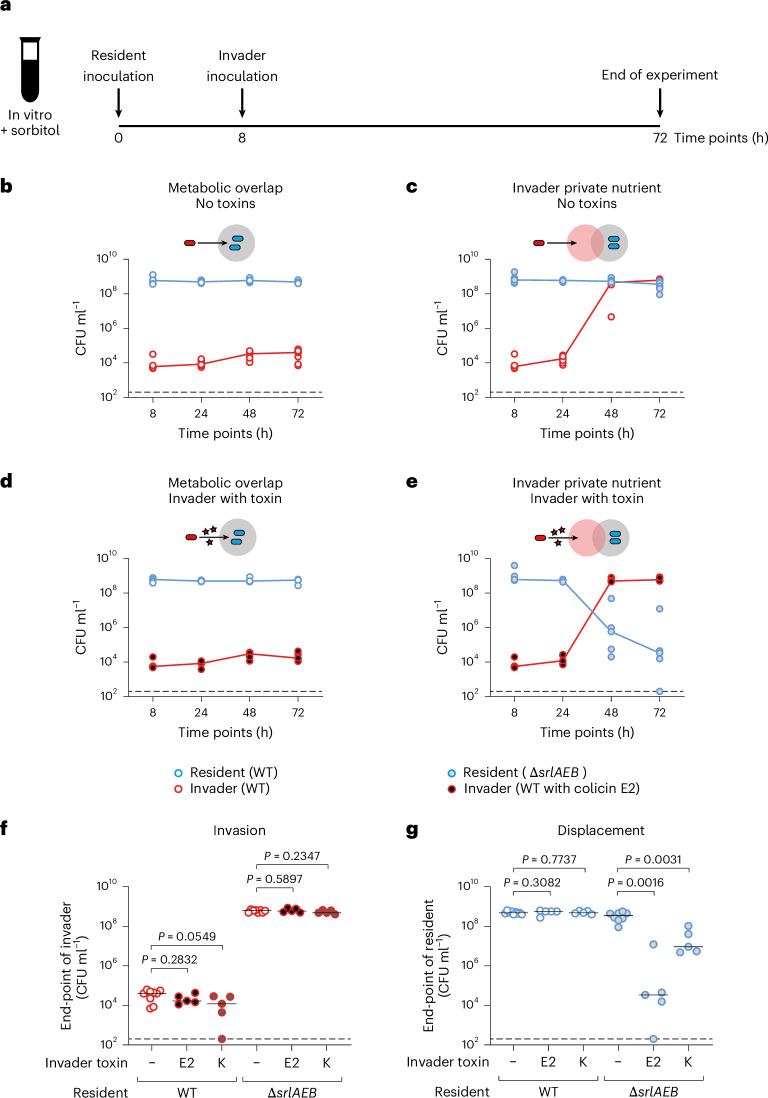
Strain displacement occurs as predicted with engineered *E. coli* strains. **a**, Scheme of invasion experiments using isogenic *E. coli* strains. LB medium is supplemented with 4% sorbitol and then inoculated with either wild-type (WT) *E. coli* or an isogenic *∆srlAEB* mutant that cannot use sorbitol. A WT *E. coli* with or without colicin E2 is inoculated 8 h later. Populations are enumerated using selective plating. **b**, Ecological invasion experiment using the scheme in **a** for scenarios modelled in Fig. 1c–f. Lines connect medians, and dashed black lines indicate the detection limits of selective plating. Here the invader does not have a private nutrient (resident is WT *E. coli*; kanamycin resistant; open blue circles) and does not have a toxin (invader is WT *E. coli*; chloramphenicol resistant; open red circles). *N* = 9 independent biological replicates, each from independent experiments. **c**, Invader has a private nutrient (resident is *E. coli ∆srlAEB*; kanamycin resistant; blue circles with grey fill) and does not have a toxin (invader is WT *E. coli*). *N* = 8 independent biological replicates each from independent experiments. **d**, Invader lacks a private nutrient (resident is WT *E. coli*) but has a toxin (invader is *E. coli* with colicin E2; chloramphenicol resistant; red circles with black fill). *N* = 5 independent biological replicates each from independent experiments. **e**, Invader has both a private nutrient (resident is *E. coli ∆srlAEB*) and a toxin (invader is *E. coli* with colicin E2). *N* = 5 independent biological replicates each from independent experiments. **f**,**g**, Same experiment as in **b**–**e**, but only the end-point values at 72 h are plotted for both the invader (**f**) and the resident (**g**). See descriptions above for sample size. Two-tailed Mann–Whitney *U* tests are used to compare population sizes when the invader does not use a toxin with when the invader uses either colicin E2 or colicin K. Population dynamics of ecological invasion experiments using colicin K are shown in Extended Data Fig. 6i,j (*N* = 5 independent biological replicates each from independent experiments for experiments with colicin K). Black lines indicate medians; dashed lines indicate the detection limits of selective plating. Source data

To manipulate the strength of nutrient competition between strains, we engineered a ∆*srlAEB* mutant of *E. coli* K12 that cannot grow on the sugar alcohol sorbitol. By then adding sorbitol in the media and using this *E. coli* ∆*srlAEB* as a resident strain, we can study the key scenario identified in the modelling in which an invading wild-type strain has a private nutrient (Fig. 2a). By contrast, if we make both the resident and the invading strain the wild type, we capture the condition of complete niche overlap. To manipulate interference competition between the two strains, we equipped the invader with colicin E2, a plasmid-borne DNAse colicin that is a potent antimicrobial protein and has been well characterized previously^19,35,41,42^. The colicin E2 plasmid is naturally carried by some *E. coli* strains and leads to both colicin production and immunity against intoxication via production of the cognate immunity protein from the same promoter. This plasmid is not transferrable without a helper plasmid, which is not present in our experiments.

**Extended Data Fig. 6:**
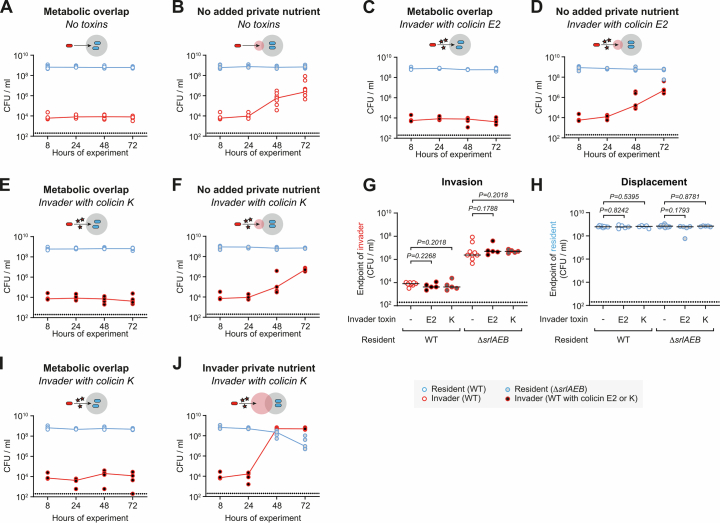
Strain displacement dynamics in the absence of supplemented sorbitol, and population dynamics in the presence of sorbitol when the invader has colicin K, related to Fig. 2. **A-H)** Ecological invasion experiments using the scheme in Fig. 2a but without 4% sorbitol. Media is inoculated with either wild-type (WT) *E. coli* or an isogenic *∆srlAEB* mutant (all resident strains kanamycin resistant; blue) that cannot use sorbitol. 8h later, a WT *E. coli* with or without colicin E2 or K is inoculated (all invader strains chloramphenicol resistant; red). Populations are enumerated using selective plating. Lines connect medians, dotted lines indicate detection limits from selective plating. **A-B)** The invader strain does not contain a toxin (*E. coli* WT; hollow red circles; *N=9* biological replicates from independent experiments). **C-D)** The invader has colicin E2 (*N=5* biological replicates from independent experiments). **E-F)** The invader has colicin K (*N=5* biological replicates from independent experiments). The resident is WT *E. coli* (hollow blue circles) in **panel A, C, E** and an isogenic *∆srlAEB* mutant (grey-filled blue circles) that cannot use sorbitol in **panel B, D, F. G-H)** Same experiment as **panel A-F**, but only the endpoint values at 72h are plotted for both the invader (**panel G**) and the resident (**panel H**). See above for sample sizes. Two-tailed Mann-Whitney U tests are used to compare population sizes when the invader does not use a toxin to when the invader uses either colicin E2 or colicin K. Black lines indicate medians, dotted lines indicate the detection limits of selective plating. **I-J)** Ecological invasion experiments using the scheme in Fig. 2a. Media is inoculated with either wild-type (WT) *E. coli* **(panel I**; hollow blue circles**)** or an isogenic *∆srlAEB* mutant (**panel J;** blue circles with grey fill; all resident strains kanamycin resistant) that cannot use sorbitol. 8h later, a WT *E. coli* with colicin K is inoculated (chloramphenicol resistant; red circles with black fill). Populations are enumerated using selective plating. Lines connect medians, dotted lines indicate detection limits from selective plating. Source data

With these tools in place, we then tested the predictions of our mathematical model with invasion assays in lysogeny broth (LB) supplemented with sorbitol (Fig. 2a). As predicted by the modelling and Theorem 1 (Supplementary Information), in the absence of a private nutrient, invasion was blocked by the resident strain whether or not the invader carried the colicin antimicrobial (Fig. 2b,d,f). By contrast, in the presence of sorbitol as a private nutrient, the invading strain was able to establish itself (Fig. 2c,f). However, only when the invader had a private nutrient and it produced colicin E2 did we see strain displacement (Fig. 2e,g). A control experiment in which we did not supplement sorbitol confirms the dependency of displacement on the invader-specific private nutrient (Extended Data Fig. 6a–h). In addition, we confirmed that our results are robust for a colicin with a different killing mechanism, colicin K, which kills by forming pores in the outer membrane of the target strain^19^ (Fig. 2f,g and Extended Data Fig. 6i,j).

These first experiments therefore support our modelling predictions that strain displacement rests upon a combination of high interference competition but low resource competition.

### Principles of strain displacement predict suppression of AMR isolates

Our first set of experiments were based upon a model strain of *E. coli*, which allowed us to modulate and isolate the type and strength of ecological competition using strains of the same genetic background. However, this approach is also artificial because competing bacterial strains in natural communities will often differ more extensively across their genomes. We therefore sought to test our modelling predictions in a second series of experiments that leverage natural variation in the degree of niche overlap between strains. Here we chose to focus on the potential to displace AMR *E. coli* strains, which are currently the dominant cause of AMR-associated deaths worldwide^24^. Leveraging natural competition mechanisms such as bacteriocin production is an interesting emerging alternative to antibiotics, because these mechanisms can be much more specific and less harmful to other microorganisms in a community^21–23^. In communities such as the mammalian microbiome, depletion of microbial diversity as a result of perturbations (that is, dysbiosis) such as antibiotic treatment is frequently associated with poor health outcomes^1^.

**Fig. 3:**
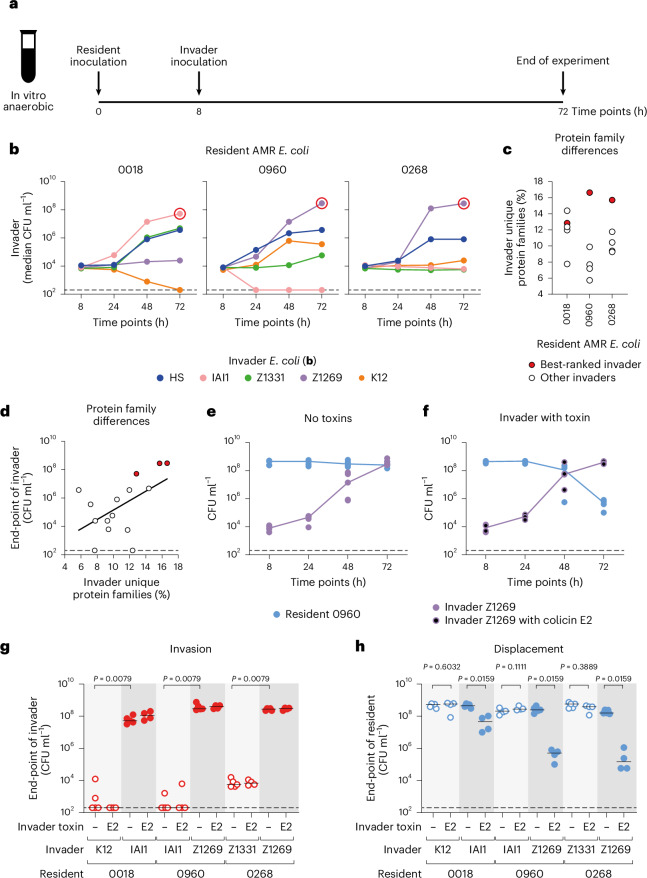
Principles of strain displacement predict suppression of AMR isolates. **a**, Scheme for invasion experiments using *E. coli* isolates. mGAM anaerobic medium in Hungate tubes is inoculated with one of three AMR isolates. After 8 h, one of five *E. coli* isolates from healthy humans is inoculated. Populations are enumerated using selective plating. **b**,Ecological invasion experiments according to the scheme in **a**. The median of *N* = 5 independent replicates for each invader (*E. coli* HS in blue, IAI1 in pink, Z1331 in green, Z1269 in purple, K12 in orange; all labelled with pACYC184 for chloramphenicol resistance) is shown at each sampled time point, and lines connect each median. Each *E. coli* isolate from healthy humans was tested for invasion into each AMR isolate (*E. coli* 0018, 0960 and 0268; ampicillin resistant). Dashed lines indicate the detection limits from selective plating. Red circles indicate the best-ranked invader for each AMR isolate. **c**, Protein family overlap between each pair of AMR isolate residents and invader isolates from healthy humans. For each pair, the percentage of protein families encoded by the invader that is not encoded by the resident is shown. For each AMR isolate, the best-ranked invader is highlighted in red (circled in **b**). **d**, Percentage of unique protein families for each strain pair (from **c**) against the end-point abundance of the invader after 72 h (data from **b**). Regression on log-transformed data and a line of best fit is shown (black line). Coefficient of determination (*R*^2^) = 0.2833, slope significantly different than 0 (Spearman’s rank correlation one tailed, *P* = 0.0230; Pearson correlation one tailed, *P* = 0.0206). The dashed line indicates the detection limit from selective plating. Strains highlighted in red in **c** are also indicated. **e**, The same data in **b** are replotted only when the resident is *E. coli* 0960 (blue) and the invader is *E. coli* Z1269 (purple). Each data point is an independent biological replicate (*N* = 5), and the lines connect the medians for each time point. **f**, The same experiment as in **e** is repeated, but *E. coli* Z1269 contains colicin E2 (colicin plasmid labelled with chloramphenicol resistance; purple circles with black fill). Each data point is an independent biological replicate (*N* = 4) determined by selective plating, and the lines connect the medians for each time point. **g**,**h**, Ecological invasion experiments as in **e** and **f** are performed for each of the best-ranked (dark grey background shade) and worst-ranked (light grey background shade) invader for each AMR isolate (*N* = 5 biological replicates from independent experiments for each strain pair without colicin E2, and *N* = 4 for each strain pair with colicin E2). The end-point abundance of the invaders is plotted in **g**, and the end-point abundance of the residents is plotted in **h**. Lines indicate medians. Two-tailed Mann–Whitney *U* tests are used to compare the population sizes of the best- and worst-ranked invaders for each AMR resident in **g** and to compare the population sizes of the resident when the invader either does or does not contain colicin E2 in **h**. In **e**–**h**, dashed lines indicate the detection limits from selective plating. Source data

For the resident strains in our experiments, we identified three clinical isolates that are sensitive to colicin E2 in vitro. Two of them are human faecal *E. coli* isolates that produce extended-spectrum beta lactamases (ESBL; 0960 and 0268), which are in a large class of highly problematic AMR strains on the World Health Organization priority list^24,40^. The third isolate is a previously characterized beta lactamase-producing urine *E. coli* isolate (0018)^17^. For the invader strains, we chose five *E. coli* symbiont strains historically isolated from the faeces of healthy humans (Supplementary Table 1). The pangenome of *E. coli* is large and metabolic diversity between strains of *E. coli* is common^39,43,44^. To test whether this variability influenced strain invasion, we first studied the ability of the five invader strains to establish in a standard gut microbiome medium (modified Gifu anaerobic medium (mGAM), buffered to human colonic pH), which had been pre-inoculated with one of the three AMR *E. coli* isolates (Fig. 3a).

Consistent with metabolic diversity among the strains, the outcome of the invasion experiments depends upon the combination of strains under study (Fig. 3b). Importantly, the experiments do not identify a single strain that is the best at invading, nor one that is best at blocking invasion. For example, *E. coli* Z1269 invades well into *E. coli* 0960 and 0268 but poorly into *E. coli* 0018. These patterns are consistent with our modelling in which invasion is dependent upon the metabolic niche available to each of the two strains. Previous work from our laboratory found that nutrient niche overlap between bacterial species in co-culture can be predicted from the degree of overlap in their protein families^17^. To explore this here, we turned to the genome sequences of the eight *E. coli* strains. We can calculate the proportion of protein families encoded by an invading strain that are not encoded by a given resident strain for each pairwise combination. Our overlap calculations reveal a significant positive relationship between the genomic difference (that is, predicted nutrient niche difference) between strains and the ecological success of the invader (Fig. 3c,d). This result is again consistent with the modelling prediction that invasion is enabled by metabolic niche diversity between an invader and resident strains, which is further supported below where we turn phenotypic data on the carbon sources that different strains and species use.

**Extended Data Fig. 7:**
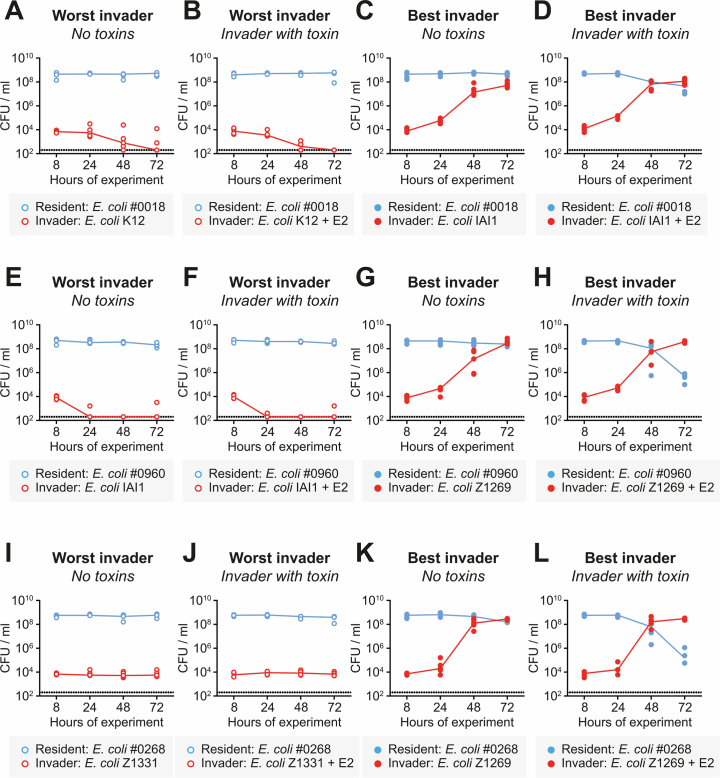
Population dynamics of ecological invasion experiments of AMR *E.* ***coli*** **strains in** Fig. 3g-h Ecological invasion experiments using scheme in Fig. 3a. The endpoint abundances of resident strains (blue) and invader strains (red) for each competition are plotted in Fig. 3g-h. Lines connect medians, dotted lines indicate detection limits from selective plating. *N=4-5* biological replicates from independent experiments for each competition. For each AMR *E. coli* isolate, the identified best and worst invader (from Fig. 3b) is tested for its ability to displace the AMR *E. coli* resident when it does or does not produce colicin E2. **a-d)** invasions when *E. coli* 0018 is the resident. **E-H)** invasions when *E. coli* 0960 is the resident. **I-L)** invasions when *E. coli* 0268 is the resident. **A-B)** Worst invader (*E. coli* K12) without **(panel A)** and with **(panel B**) colicin E2. **C-D)** Best invader (*E. coli* IAI1) without **(panel C)** and with **(panel D**) colicin E2. **E-F)** Worst invader (*E. coli* IAI1) without **(panel E)** and with **(panel F**) colicin E2. **G-H)** Best invader (*E. coli* Z1269) without **(panel G)** and with **(panel H**) colicin E2 (same data as Fig. 3e-f). **I-J)** Worst invader (*E. coli* Z1331) without **(panel I)** and with **(panel J**) colicin E2. **K-L)** Best invader (*E. coli* Z1269) without **(panel K)** and with **(panel L**) colicin E2. Source data

We next investigated what happens when an invader strain is also capable of interference competition. As a test case, we equipped one of our invader strains (Z1269) with colicin E2 and performed the same invasion assay into *E. coli* 0960 as before. As expected from previous results, without the antimicrobial, Z1269 invaded well but did not displace the resident strain *E. coli* 0960 (Fig. 3e). By contrast, with the antimicrobial, Z1269 could both invade and markedly reduce the number of the AMR strain (Fig. 3f). We then extended the experiments to include the best-ranked invader and worst-ranked invader for each of the three AMR *E. coli* isolates. In all cases, when a best-ranked invading strain produced colicin E2, it was able to both invade (Fig. 3g) and suppress the AMR *E. coli* isolate (Fig. 3h and Extended Data Fig. 7). Importantly, and consistent with Theorem 1 (equation (2) and Supplementary Information), all the worst-ranked invading strains were unable to invade whether or not they carried the antimicrobial colicin (Fig. 3g). In summary, in competitions between natural isolates of *E. coli*, we find again that strain displacement rests upon the combination of expected low nutrient competition and high interference competition.

### Overcoming nutrient blocking enables strain displacement in diverse bacterial communities

**Fig. 4:**
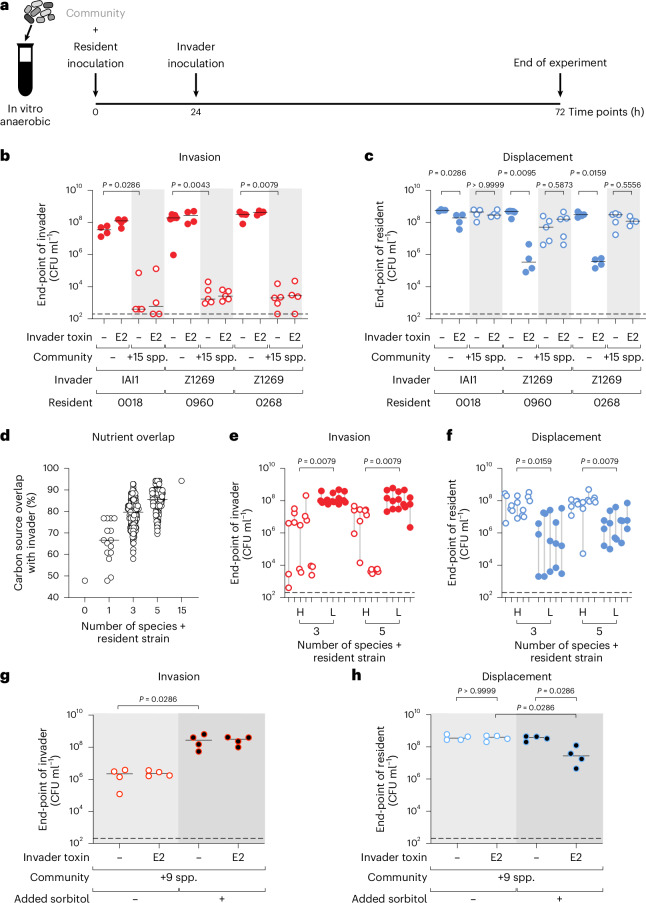
Overcoming nutrient blocking enables strain displacement in diverse bacterial communities. **a**, Scheme for community invasion experiments. **b**,**c**, Experiments as in **a** are performed for the best-ranked invader for each AMR isolate (from Fig. 3b) in the absence (white background; red filled circles) or presence (light grey background shade; open red circles) of a 15-member community (+15 spp.; see Supplementary Table 1 for strains used). For each strain pair, the invader either contains colicin E2 or remains the wild-type *E. coli* invader. *N* = 4 biological replicates from independent experiments for all combinations, with the exception of *N* = 5 for four combinations (Z1269 and 0960 in the presence of 15 species both when Z1269 does and does not have colicin E2; Z1269 and 0268 when Z1269 does not have a toxin, both in the absence of a 15-species community), and *N* = 6 for the strain pair of Z1269 and 0960 in the absence of a community and colicin E2. The end-point abundance of the invaders is plotted in **b**, and the end-point abundance of the residents is plotted in **c**. Lines indicate medians. Two-tailed Mann–Whitney *U* tests are used to compare the population sizes of invaders in **b** and to compare the population sizes of the resident in **c**. Dashed lines indicate the detection limits from selective plating. **d**, In silico prediction of carbon-source overlap of the resident AMR *E. coli* 0960 and the community to the invader *E. coli* Z1269. Each circle represents a different community and all possible combinations of the 15 species in addition to the focal strain pair at each diversity level is plotted (*N* = 1, *N* = 15, *N* = 455, *N* = 3,003, *N* = 1 for 0, 1, 3, 5 and 15 additional species, respectively). AN Biolog assays are used to determine carbon source overlap of each individual strain (Extended Data Fig. 9a), and predicted carbon-source overlap of the community is calculated using an additive approach as done in ref. ^17^. **e**,**f**, The effects on invasion and displacement of five communities with the highest (H; open red circles) and lowest (L; filled red circles) overlap to the invader at diversity levels of both three and five species in addition to the focal *E. coli* strains were tested (communities identified in **d**). For each of the five communities in each group, *N* = 3 biological replicates from independent experiments are shown and grey lines connect the highest and lowest point of the three replicates. Dashed lines indicate the detection limits from selective plating. The end-point (72 h) abundance of invader *E. coli* Z1269 is plotted in **e**, and the end-point abundance of resident *E. coli* 0960 is plotted in **f**. Two-tailed Mann–Whitney *U* tests are used to compare the end-point of the invader in **e** with the end-point of the resident in **f**. In all cases, the median value of the three replicates for each community is used. This means that Mann–Whitney *U* tests use community medians for the comparisons (*N* = 5 communities for each). **g**,**h**, Nutrient supplementation experiment in the presence of a diverse community. The AMR isolate *E. coli* 0018 *∆srlAEB* is the resident and embedded in a community of 9 species that cannot use sorbitol (+9 spp.; see Supplementary Table 1 for strains used; inability to use sorbitol defined in Extended Data Fig. 9a). One-half mGAM medium without (light grey background shade; open circles) or with 1% sorbitol (dark grey background shade; circles with black fill) is used as the medium. The invader strain is *E. coli* IAI1 with or without colicin E2. *N* = 4 biological replicates from independent experiments. The end-point (72 h) abundance of the invader is plotted in **g**, and the end-point abundance of the resident is plotted in **h**. Two-tailed Mann–Whitney *U* tests are used to compare the end-point abundance of the invader in **g**, and to compare the end-point abundance of the AMR *E. coli* resident in **h**. Source data

**Extended Data Fig. 8:**
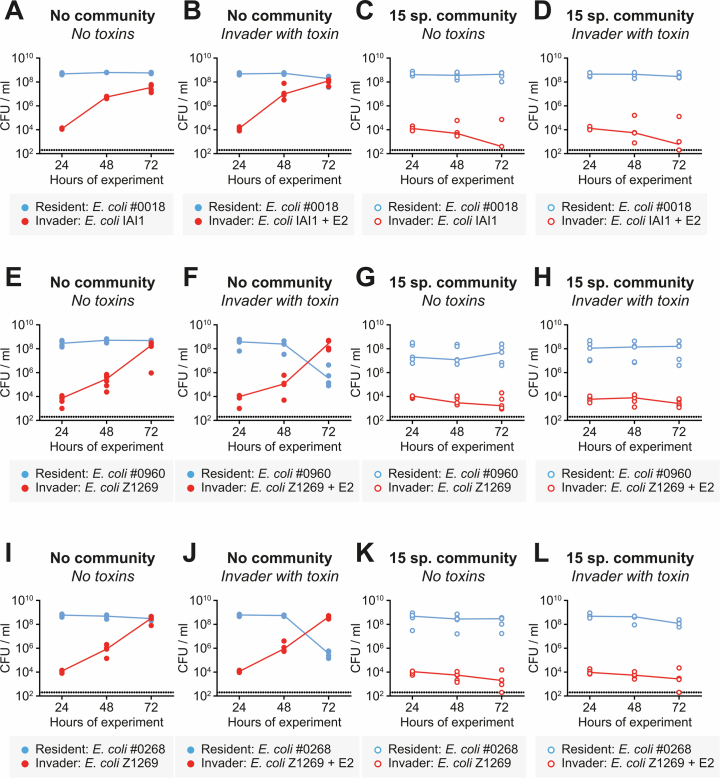
Population dynamics of community invasion experiments of AMR *E.* ***coli*** **strains in** Fig. 4b-c Community invasion experiments using scheme in Fig. 4a. The endpoint abundances of resident strains (blue) and invader strains (red) for each competition are plotted in Fig. 4b-c. Lines connect medians, dotted lines indicate detection limits from selective plating. *N=4-6* biological replicates from independent experiments for each competition. For each AMR *E. coli* isolate, the identified best invader (from Fig. 3b) is tested for its ability to displace the AMR *E. coli* resident when it does (indicated with “+E2” in the panel) or does not produce colicin E2, in the presence or absence of a 15-species community. **A-D)** invasions when *E. coli* 0018 is the resident and *E. coli* IAI1 is the invader. **E-H)** invasions when *E. coli* 0960 is the resident and *E. coli* Z1269 is the invader. **I-L)** invasions when *E. coli* 0268 is the resident and *E. coli* Z1269 is the invader. **A-B, E-F, I-J)** Two focal *E. coli* strains compete in the absence of a diverse community. **C-D, G-H, K-L)** Competitions between focal *E. coli* are embedded within a diverse community of 15 species (see Table S1 for strains). Source data

We have so far studied competition scenarios in which an invading strain faces a resident strain of the same species in isolation. However, many microbial communities contain a diversity of species. These species can interact with one another in ways that affect ecological outcomes^8,45^. Therefore, we next performed *E. coli* invasion experiments in the presence, or absence, of a diverse community, focusing on the best-ranked invading strain for each AMR *E. coli* isolate. For the community, we chose a community of 15 species that we had previously characterized and contains phylogenetically distinct representatives of common human gut symbionts^17^. We grew each symbiont species separately, assembled them in 15-species communities along with the resident AMR *E. coli* isolate and then challenged the resulting community with the best-ranked invader (Fig. 4a). Note that colicin E2 is a narrow-spectrum bacteriocin, meaning that the community is not expected to be directly affected by its action^23^. As expected from the last set of experiments, when the community was absent, all invaders established themselves in the presence of the resident (Fig. 4b) and suppressed its numbers (Fig. 4c). However, the outcome changed in the presence of the community. Adding the 15-species community always blocked the invader from establishing (Fig. 4b) and consequently also blocked any displacement (Fig. 4b,c and Extended Data Fig. 8).

**Extended Data Fig. 9:**
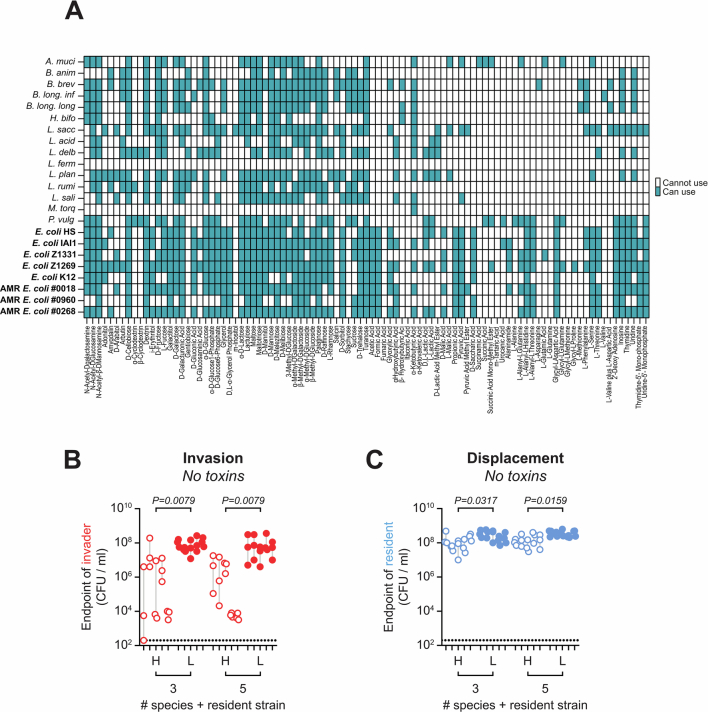
Individual carbon source utilization profiles of strains, and community experiments in Fig. 4e-f but the invader does not have a toxin. **A)** All 95 individual carbon sources tested in the Biolog AN MicroPlates are listed along the x-axis. Each of the 15 symbiont strains were previously tested^17^; see Table S1 for full species and strain names. In addition, the five *E. coli* isolated from healthy humans and three AMR *E. coli* isolates are tested. Nutrients shaded in blue were defined as being able to use the indicated nutrient whereas those in white were defined as not able to use the nutrient (see **methods**). Data used to apply thresholding on Abs_590nm_ measurements to define nutrient utilization capabilities comes from the median of three independent biological replicates for each strain. **B-C)** Effect of communities on invasion of the invader and displacement of the resident if the invader does not have a toxin. The same communities used in Fig. 4e-f are used for this control experiment (scheme Fig. 4a), but now the invader *E. coli* Z1269 does not produce colicin E2. The effects on invasion and displacement of 5 communities with the highest (H; hollow red circles) and lowest (L; filled red circles) overlap to the invader at diversity levels of both three and five species in addition to the focal *E. coli* strains were tested (communities identified in Fig. 4d). For each of the 5 communities in each group, *N=3* biological replicates from independent experiments are shown and grey lines connect the highest and lowest point of the three replicates. Dotted lines indicate the detection limits from selective plating. The endpoint (72h) abundance of the invader *E. coli* Z1269 is plotted in **panel A** and the endpoint abundance of the resident *E. coli* 0960 is plotted in **panel B**. Two-tailed Mann-Whitney U tests are used to compare the endpoint of the invader in communities with the highest or lowest predicted carbon-source overlap in **panel A** and the endpoint of the resident in **panel B**. In all cases, the median value of the three replicates for each community is used. This means that Mann-Whitney U tests use community medians for the comparisons (*N=5* communities for each). Source data

This impact of the community is consistent with previous work showing that diverse communities can block invasion of pathogens collectively. This process, known as nutrient blocking, occurs whenever there is sufficient overlap in the nutrient utilization abilities of the community as a whole compared with the invading strain^17^. To explore the importance of nutrient blocking, we focused on the case of the invading strain *E. coli* Z1269 and the AMR *E. coli* isolate 0960. We use previously published data on the nutrient utilization abilities of the 15 symbionts from Biolog AN MicroPlates (Extended Data Fig. 9a)^17^ and also collected Biolog data for the *E. coli* strains for the purposes of this study, which revealed that *E. coli* 0960 could metabolize only around half of the carbon sources on the plate used by *E. coli* Z1269 in these experimental conditions (Fig. 4d). Not all carbon sources in our growth media are found on the Biolog plates, and vice versa. Nevertheless, previous work found that the Biolog plates are a good proxy for general carbon source overlap between strains^17^. We computationally assembled all possible combinations of 1, 3 and 5 species for the other 15 symbionts and calculated the carbon source overlap between these communities (when they also contain the *E. coli* resident) and the invading strain *E. coli* Z1269. As the diversity of the community increases, we observe that the probability of community-conferred carbon source overlap with the invading strain is higher, again consistent with previous work^17^ (Fig. 4d). Importantly, when all 15 species are present, the community can metabolize nearly all tested carbon sources compared with the invading strain. This suggests that high metabolic overlap explains why the 15-species community blocked invasion and consequent displacement in our experiments (Fig. 4b,c).

We further tested the importance of nutrient blocking by using our carbon-source overlap calculations to identify three- and five-species communities which, when added to the resident *E. coli* strain, have either the highest or lowest overlap with the invading strain. Specifically, for each level of diversity, we chose five communities that had the highest overlap (H) and five communities that had the lowest overlap (L) and then performed the same experiment as before (Fig. 4a). As predicted, the invader invaded effectively when the community was predicted to have low overlap relative to the invader, but failed to invade reliably when the community was predicted to have high overlap (Fig. 4e). Importantly, this led to efficient displacement of the resident in the low-overlap communities but not in the high-overlap ones (Fig. 4f). Moreover, displacement never occurred when the invader lacked the ability to make colicin antimicrobials (Extended Data Fig. 9b,c). These experiments, therefore, again support the importance of nutrient blocking for strain displacement. Specifically, for displacement to occur in our experiments, an incoming strain must be able to overcome both nutrient competition from conspecific strains in its niche as well as collective nutrient blocking that involves multiple members of the resident community.

**Extended Data Fig. 10:**
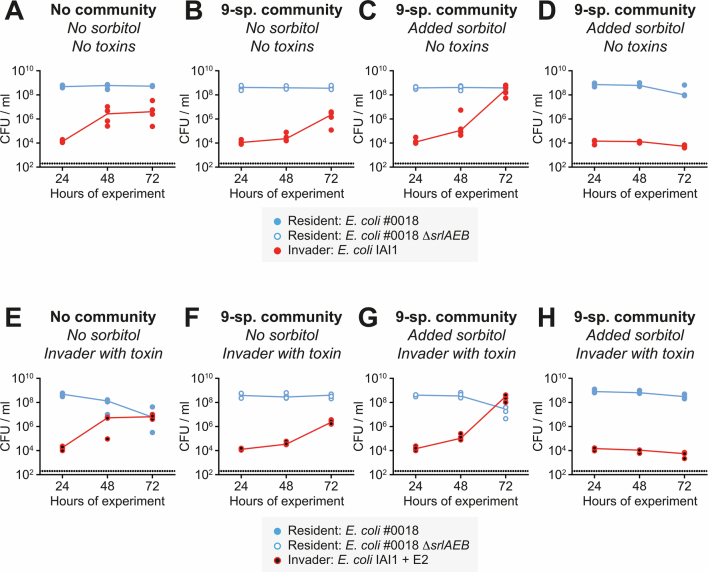
Population dynamics of sorbitol supplementation experiments in diverse communities in Fig. 4g-h, plus additional controls. Nutrient supplementation community invasion experiments according to the scheme in Fig. 4a. Lines connect medians, dotted lines indicate the detection limits from selective plating. *N=*3-4 biological replicates from independent experiments for each competition. **A-D)** *E. coli* IAI1 WT is used as the invader (red filled circles). **E-H)** *E. coli* IAI1 with colicin E2 is used as the invader (red circles with black fill). **A,E)** The AMR isolate *E. coli* 0018 is the resident (blue filled circles), sorbitol is not added to the media, and there are no additional symbiont strains in the competition. **B,F)** The AMR isolate *E. coli* 0018 *∆srlAEB* is the resident (blue hollow circles), sorbitol is not added to the media, and the competition is embedded in a community of 9 species that cannot use sorbitol (+9sp., see Table S1 for strains used; inability to use sorbitol defined in Extended Data Fig. 9A). **C,G)** Sorbitol is supplemented to the media (1% sorbitol), but otherwise the experimental set-up is the same as in **panel B,F**. **D,H)** The AMR isolate *E. coli* 0018 WT is the resident, sorbitol is added to the media (1%), and the competition is embedded in a community of 9 species that cannot use sorbitol. Source data

As a final test of our ideas, we asked whether one can overcome nutrient blocking of an invading strain and drive strain displacement by supplementing with a private nutrient. We ran a version of our invasion experiment using a community of nine species that cannot metabolize sorbitol. We used genome editing to generate a ∆*srlAEB* mutant, which cannot use sorbitol, in the background of the AMR *E. coli* isolate 0018 and used this as the resident strain. As a result, no member of the resident community can use sorbitol in this experiment. *E. coli* IAI1 was the invading strain with colicin E2 for interference competition. This strain normally invades well and displaces *E. coli* 0018 in the absence of a community (Fig. 4b,c). By contrast, in the presence of the nine-species community, we observe nutrient blocking and poor invasion (Fig. 4g), without an observable impact on *E. coli* 0018 (Fig. 4h). However, as predicted, the addition of sorbitol as a private nutrient drives an increase in invasion by ~100-fold (Fig. 4g) and the suppression of the AMR *E. coli* target strain within the community of 9 species (Fig. 4h). We confirmed that this result is dependent on sorbitol not being consumed by the community: when *E. coli* 0018 can use sorbitol (*E. coli* 0018 wild type used as the resident), *E. coli* IAI1 could no longer invade, even under sorbitol supplementation (Extended Data Fig. 10). In summary, the results from diverse communities of bacteria again support our modelling predictions that competitive strain displacement rests upon weakening nutrient competition in order that an invading strain can establish and engage in interference competition that suppresses a resident strain.

## Discussion

There is currently a great interest in microbial communities and what determines their composition. Here we have studied the process of strain displacement using a combination of ecological modelling and experimental tests. Our general mathematical model of microbial communities identifies a specific scenario that enables strain displacement events via ecological competition. Firstly, the invading strain needs to experience relatively low levels of competition to establish in a community. Secondly, the strain needs to engage in strong interference competition to remove a resident strain from its shared niche (Fig. 1). We verified these predictions using *E. coli* as a test case, by genetic engineering that allowed us to manipulate both resource and interference competition (Fig. 2), and with competitions between natural strains of *E. coli* (Fig. 3). Finally, we studied the impacts of diverse communities on the outcome of strain displacement. An invading strain must be able to overcome nutrient competition not only with its niche competitor but also with the wider community to displace the niche competitor (Fig. 4). In our experiments, this outcome was achieved by using a private nutrient that is untouched by the community, but another potential route for a strain to invade would be to use a partially consumed nutrient more efficiently than any member of the resident community.

Our findings fit well with previous work that has studied either resource or interference competition. Nutrient competition is known to be an important determinant of the ability of a strain to establish itself in a community^10,14–17,32^. For interference competition, there is considerable evidence that the benefits of bacteriocin production are density dependent^23,36^, which we see in both our models and experiments (Figs. 1–4). Here, we also have identified a strong interaction between these two forms of competition. The density-dependent benefits of antimicrobials can be a major issue for an invading strain experiencing strong nutrient competition because this can prevent a strain from establishing itself well enough to make use of antimicrobials.

The impacts of weapons at low frequency or density may also be influenced by spatial structure, such as occurs in biofilms^46^, as spatial structure can improve the effectiveness of bacterial weapons. The importance of spatial structure in the mammalian gut, where peristalsis actively mixes gut contents, is a matter of active study^47^, but other settings, such as oral biofilms, are highly structured^48^. Nevertheless, our theory predicts that it is access to a private nutrient, rather than interference competition, which enables strains to invade an existing community, be it well mixed or spatially structured (Fig. 1 and Extended Data Fig. 5). Therefore, we anticipate that our key conclusion that nutrient competition is central to invasion will hold across many environments. Indeed, recent work has shown that the benefits of both contact and diffusing weapons are strongly frequency dependent under conditions of spatial structuring. Consistent with our findings, this work found that benefits of weapons are typically greatest once strains have has reached a certain density or frequency, an effect that is particularly strong for diffusing toxins^34^. Our findings are also likely to apply to cases in which a pathogenic strain invades a community and displaces a non-pathogenic resident. For example, *Salmonella enterica* subsp. *enterica* serovar Typhimurium uses its metabolism to grow to sufficient densities^49^, at which point it triggers inflammation and uses antibacterial toxins to target *E. coli* competitors^20^.

If interference competition is so important for the ability of one strain to suppress another, why then do not all bacterial strains evolve to carry weapons^18^? Bacterial weapons such as colicins can be very costly to use^41^, and if a resident strain uses few overlapping nutrients, the best evolutionary strategy may be to coexist rather than attack. The question of how interference competition and metabolism evolve in the face of variable nutrient competition is an interesting question for future work. However, in some cases, strain turnover is likely to occur without a role for bacterial weapons. If nutrient conditions change, strain invasion and turnover can occur simply because the nutrients previously available to a resident are no longer present. We anticipate, therefore, that strain turnover in natural systems occurs both in this passive manner as well as via active competition and displacement as we have described here^30,50,51^.

The study of strain displacement has implications for biotherapeutic strategies that seek to change the composition of microbial communities. Strains of AMR *E. coli* or *Klebsiella* sp. are common residents of the gut microbiome but rarely cause disease in the gut itself. However, they can spread AMR^24,40^ and cause deadly diseases elsewhere in the body^52^. The prospect of removing these strains from the gut microbiome, therefore, is an attractive one. While we have focused on targeting AMR strains, their antibiotic resistance itself is not expected to influence strain replacement in the absence of clinical antibiotics. Instead, the key to displacement will lie in finding a biotherapeutic strain that does not overlap too greatly with the target in the nutrient utilization profile. This outcome can be achieved by supplementing a microbiome with a private nutrient for a biotherapeutic strain (Fig. 4), as illustrated for *B. thetaiotamicron* previously^33^. Alternatively, one might try to introduce a high enough density of a biotherapeutic strain to enable it to use a bacterial weapon immediately, although this may be challenging in practice (Extended Data Fig. 4).

Such strategies all assume that one can identify a bacterial weapon that a biotherapeutic strain can use against a target strain. Interference competition is found in many species of bacteria, including many gut symbionts such as *Bacteroides* spp.^18,53^, which suggests that the use of bacterial weaponry in this way is feasible. One caveat from our data is that the negative impacts on a resident strain from the antimicrobial are sometimes quite modest, seemingly too modest to be used as a biotherapeutic strategy. However, our experiments are relatively short and that these impacts are likely to be much greater over longer periods, which allows a toxin-producing strain more time to displace a resident. The models also make clear that another route to increasing the degree of displacement is via the development of more potent toxins against a particular target strain, which is another interesting avenue for future work.

Despite the ecological complexity of microbial communities, our study suggests that there are ultimately simple rules underlying what makes a highly competitive microorganism. These principles can be applied in strategies that seek to rationally manipulate microbial communities by introducing beneficial strains, removing problematic strains or both.

## Methods

### Mathematical model

Our general theory of ecological invasions is based upon a consumer-resource model (equation (1), Results), meaning that the abundance of all strains is determined by nutrients in the system, along with any toxins they may encounter. Although the abundance of strains can also be modulated by density-dependent regulation^54^, this will take effect only once cells reach a density ultimately determined by the nutrients or toxins in our model. Therefore, we do not consider density-dependent regulation explicitly here.

The Supplementary Text provides further details of the general model including the proof of the key Theorem 1 introduced in Results. Here we provide an overview of the model with a single resident and invader strains that we use to illustrate the general theory and to generate Fig. 1 and Extended Data Figs. 1 and 2. For the model, we consider a resident strain with abundance *N*_R_ that competes with an invader strain of abundance *N*_I_ over shared nutrients with concentration *x* in a continuous culture. Moreover, we assume that each strain can also use private nutrients with concentrations *x*_R_ (or *x*_I_ for the invader private nutrient) and that the invader strain can target the resident strain with a toxin of concentration *y*. As bacteria have a finite proteome budget that is partitioned among different physiological functions^55^, we introduce the toxin investment parameter *z* ∈ [0,1] for the invader strain as the relative investment into proteome associated with toxin production as opposed to nutrient metabolism. Correspondingly, we assume that the toxin is produced at a rate *gz*, diluted at rate *d*, taken up by the resident at maximal rate *s* with a Monod constant *K* and has maximal potency *p* to decrease the growth rate of the resident population. Furthermore, we assume that the invader (respectively, the resident) uses the private nutrients at a maximal consumption rate (1 − *z*)*C*_I_, a maximal growth rate (1 − *z*)*R*_I_ and a Monod constant *K*_I_ (respectively, *C*_R_, *R*_R_ and *K*_R_) and the shared nutrients at a maximal consumption rate (1 − *z*)*c*_I_, a maximal growth rate (1 − *z*)*r*_I_ and a Monod constant *k*_I_ (respectively, *c*_R_, *r*_R_ and *k*_R_). We assume that the strains grow at rates given by a generalized Monod equation^56,57^ and are diluted at rate *δ*. Finally, we assume that the nutrients are diluted at rate *D* and supplied at rates *m* (shared nutrients), *m*_R_ (private nutrients of the resident strain) and *m*_I_ (private nutrients of the invader strain). With these assumptions, the resulting general consumer-resource dynamics in equation (1) (Results) reduces to

3 $$\,\begin{array}{l}{\dot{N}}_{{\rm{R}}}={N}_{{\rm{R}}}\left(\frac{{r}_{{\rm{R}}}x/{k}_{{\rm{R}}}+{R}_{{\rm{R}}}{x}_{{\rm{R}}}/{K}_{{\rm{R}}}}{1+x/{k}_{{\rm{R}}}+{x}_{{\rm{R}}}/{K}_{{\rm{R}}}}-\frac{py}{y+K}-\delta \right)\\ {\dot{N}}_{{\rm{I}}}\,=\,{N}_{{\rm{I}}}\left((1-z)\frac{{r}_{{\rm{I}}}x/{k}_{{\rm{I}}}+{R}_{{\rm{I}}}{x}_{{\rm{I}}}/{K}_{{\rm{I}}}}{1+x/{k}_{{\rm{I}}}+{x}_{{\rm{I}}}/{K}_{{\rm{I}}}}-\delta \right)\\ \dot{x}\,=\,m-Dx-{N}_{{\rm{R}}}\frac{{c}_{{\rm{R}}}x/{k}_{{\rm{R}}}}{1+x/{k}_{{\rm{R}}}+{x}_{{\rm{R}}}/{K}_{{\rm{R}}}}-{N}_{{\rm{I}}}(1-z)\frac{{c}_{{\rm{I}}}x/{k}_{{\rm{I}}}}{1+x/{k}_{{\rm{I}}}+{x}_{{\rm{I}}}/{K}_{{\rm{I}}}}\\ {\dot{x}}_{{\rm{R}}}={m}_{{\rm{R}}}-D{x}_{{\rm{R}}}-{N}_{{\rm{R}}}\frac{{C}_{{\rm{R}}}x_{{\rm{R}}}/{K}_{{\rm{R}}}}{1+x/{k}_{{\rm{R}}}+{x}_{{\rm{R}}}/{K}_{{\rm{R}}}}\\ {\dot{x}}_{{\rm{I}}}={m}_{{\rm{I}}}-D{x}_{{\rm{I}}}-{N}_{{\rm{I}}}(1-z)\frac{{C}_{{\rm{I}}}{x}_{{\rm{I}}}/{K}_{{\rm{I}}}}{1+x/{k}_{{\rm{I}}}+{x}_{{\rm{I}}}/{K}_{{\rm{I}}}}\\ \dot{y}=zg{N}_{{\rm{I}}}-dy-{N}_{{\rm{R}}}\frac{sy}{y+K}.\end{array}$$

We simulated this system of equations numerically with the explicit Runge–Kutta method (time step Δ*t* = 1, maximal time *t* = 300) in Python to explore invasion dynamics (Fig. 1c–f) and examined its steady states by a combination of analytical techniques (Supplementary Text) and numerical root finding with the SciPy package^58^ to identify regions where the invader strains can successfully invade and displace the resident strain (Fig. 1b and Extended Data Fig. 1). In particular, the displacement boundary in Fig. 1b and Extended Data Fig. 1f is obtained by performing a binary search over the toxin investment (or toxin potency for Extended Data Fig. 1a,k) domain to determine a critical value for the existence of a non-negative fixed point to equation (3) with *N*_I_,*N*_R_ > 0. The hybrid method from the SciPy package^58^ with the initial estimation of *N*_I_ = *N*_R_ = *N*′, *x* = *x*′, *x*_I_ = *x*_R_ = *x*_R_′, 𝑦 = 0 (with values *N*′, *x*′ and *x*_R_′ given by Supplementary Equation (11) in Supplementary Text) is used to determine the existence of this coexistence fixed point.

### Bacterial strains

All strains used in this study are listed in Supplementary Table 1, all plasmids used in this study are listed in Supplementary Table 2 and all primers used to construct or verify strains are listed in Supplementary Table 3. For strains grown under aerobic conditions (*E. coli* strains), LB (Fisher Scientific) medium was used containing the appropriate antibiotic (100 µg ml^−1^ ampicillin, 50 µg ml^−1^ kanamycin, 15 µg ml^−1^ chloramphenicol). For strains grown under anaerobic conditions (5% H_2_, 5% CO_2_, 90% N_2_, <20 ppm O_2_), mGAM (Nissui Pharmaceuticals) broth buffered to pH 6.2 with 100 mM 2-(*N*-morpholino)ethanesulfonic acid (MES; Sigma-Aldrich) was used. To ensure that anaerobic conditions were maintained, the redox indicator dye resazurin (100 µg l^−1^ medium; Sigma-Aldrich) was added to the medium. All strains were grown at 37 °C shaking at 220 rpm. Strains were stored in glycerol stocks at −70 °C with a final concentration of 25% glycerol.

The strains chosen to make up the 15-member community are important members of the human gut microbiota that cover key phylogenetic groups and were previously characterized^17^. All strains used in this study are either previously sequenced or sequenced in the scope of this study.

### Genetic engineering of bacterial strains

Plasmids and genetic constructs were transformed by either electroporation or by heat-shock. For electroporation, 5 ml of overnight culture grown in LB was concentrated and washed 3× with ice-cold Milli-Q water. Subsequently, 2 µl of plasmid (prepped using a QIAprep Spin Miniprep Kit, Qiagen) was mixed with 100 µl of concentrated cells and electroporated (1.8 kV; 1 mm gap cuvettes). For heat-shock, overnight cultures of cells were subcultured until OD 0.5, washed twice with ice-cold 100 mM CaCl_2_ containing 15% glycerol and then concentrated. The cells were aliquoted and either frozen at −70 °C for later use or mixed with 2–10 µl plasmid on ice for 30 min. Cells incubated with the plasmid were then heat-shocked for 40 s at 42 °C and then put back on ice for 3 min. After either type of transformation, cells were recovered in 1 ml LB (1 h of shaking at 37 °C, or 30 °C for pKD46) before being plated on the appropriate selective LB media.

To create gene deletions or insertions in *E. coli* K12 strains, lambda red engineering was used^59^. PCR (KOD high-fidelity DNA polymerase, Merck) was used to amplify chloramphenicol or kanamycin resistance cassettes from pKD3 or pKD4, respectively, with homology to the target gene locus. The target strain containing the pKD46 helper plasmid (BZB1011 pKD46) was cultured overnight at 30 °C with 100 µg ml^−1^ ampicillin and subcultured (1/100) in LB for 2 h. The lambda red system was then induced by adding 10 mM L-arabinose for 1 h. Then, 5 µl of the PCR template was electroporated into the induced cells. Mutants were verified using PCR.

To transform natural colicin plasmids (pColE2-P9 or pColK-K235) into *E. coli* K12 strains, isolated colicin plasmids were electroporated into the target strain and mutants were selected for on colicin supernatant. To prepare supernatant-containing plates, strains containing the desired colicin were grown overnight in LB with 0.25 µg ml^−1^ mitomycin C (Merck) and centrifuged, and the supernatant was filtered with a 0.22-µm filter. Before selective plating, 200 µl of the supernatant was spread onto an LB plate. Candidate transformants were confirmed as colicin producers by determining that the supernatant produced by these transformants could inhibit wild-type *E. coli* K12 but not the colicin-containing ancestral strain using the same method of supernatant preparation and streaking of the target strains on the plate.

To transform colicin E2 into natural *E. coli* isolates (*E. coli* HS, IAI1, Z1269 and Z1331), a chloramphenicol resistance marker was cloned into pColE2-P9 yielding pColE2-Cm_v2, and electroporated into target strains using selection on chloramphenicol. To create pColE2-Cm_v2, the chloramphenicol resistance cassette was amplified from pHis-MBP-T_H using primers cmv2_fw and cmv2_rv, and the pColE2-E9 vector was amplified using primers e2_cmv2_fw and e2_cmv2_rev. Fragment and vector were joined using Gibson Assembly (NEBuilder HiFi DNA Assembly, New England Biolabs) according to the manufacturer’s instructions. Using heat-shock, 5 µl of the assembled plasmids was transformed into *E. coli* DH5α and selected for with chloramphenicol (15 µg ml^−1^). Plasmids from successful transformants were isolated using miniprep and confirmed via Sanger sequencing using primers e2_1-7 and cmv2_fw.

To engineer natural *E. coli* isolates (*E. coli* 0018 ∆*srlAEB*), a suicide vector method with dual negative selection was used^60^. Briefly, 700 base pairs upstream and downstream of the region to be deleted was amplified using PCR (KOD high-fidelity DNA polymerase, Merck). pFOK was digested with BamHI-HF and EcoRV-HF (New England Biolabs) for 1 h at 37 °C and gel extracted. Subsequently, 50 ng of digested pFOK was mixed with 2–3-fold molar excess of each of the flanking regions and assembled using Gibson Assembly (NEBuilder HiFi DNA Assembly, New England Biolabs) at 50 °C for 60 min. The assembled mix was transformed into *E. coli* EC100D *pir-116* using heat-shock. Resulting clones were verified for the correct insert at the multiple cloning site using PCR and Sanger sequencing (Source Biosciences) using primers oOPC614 and oOPC615. Suicide vector plasmids were then isolated and then transformed into the DAP auxotroph *E. coli* JKe201 using heat-shock (100 µM DAP supplemented to the media). For integration of the suicide vector into the target strain, both the JKe201 donor strain containing the suicide vector and the target strain were grown overnight without antibiotics and then mixed at a 1:1 ratio, concentrated, spotted on an LB plate and allowed to grow for at least 6 h at 37 °C. To select for merodiploids, the mixture was streaked on LB with 50 µg ml^−1^ kanamycin to select for suicide vector integration. Merodiploids were then streaked on LB without salt containing 20% sucrose and 0.5 µg ml^−1^ anhydrous tetracycline and incubated in the dark at 28 °C for at least 24 h. Deletion mutants were then screened with PCR.

### Isogenic *E. coli* invasion experiments (aerobic)

Overnight cultures of the competing *E. coli* strains were inoculated from single colonies in LB with the appropriate antibiotics. The medium used for the invasion experiments is ½ LB (diluted with Milli-Q water) or ½ LB + 4% sorbitol (diluted from 20% sorbitol with Milli-Q water) buffered to pH 6.2 with 50 µM MES. The LB medium was buffered to pH 6.2 with 100 µM MES and then autoclaved, before mixing with sterile-filtered sorbitol and autoclaved Milli-Q water. Aliquots of 3 ml medium in test tubes were used for the assay. The resident strains were washed 2× with PBS, and 3 µl of washed cells was added to the medium (corresponding to 10^6^ CFU ml^−1^ starting density). After 8 h, the invader strains were removed from the incubator, washed 2× with PBS and diluted such that 10^4^ CFU ml^−1^ was added to each tube as a starting inoculum (that is, 30 µl of 10^−3^-diluted washed cells). The inocula were diluted and plated on LB with the appropriate antibiotics. At distinct time points as indicated on the figures (for example, Fig. 2), tubes were removed from the incubators; samples were collected (20 µl volume), diluted and plated on selective medium (LB plus kanamycin or chloramphenicol); and then tubes were returned to the incubator until 72 h after inoculation of the resident strain.

### Invasion experiments with *E. coli* isolates (anaerobic)

The medium used for these experiments was mGAM buffered to pH 6.2 with MES. The medium was pre-reduced in an anaerobic chamber (Coy) containing a gas composition of 5% H_2_, 5% CO_2_, 90% N_2_ and <20 ppm O_2_, and the redox indicator resazurin was used to ensure oxygen remained limited during the course of the experiment. All strains and competition experiments were grown in sealed Hungate tubes that could be sampled using needles and syringes (5 ml medium volume). Overnight cultures of *E. coli* strains in Hungate tubes with buffered mGAM were inoculated from single colonies and grown at 37 °C.

As for the aerobic invasion experiments with isogenic *E. coli*, anaerobic cultures of the resident strains were washed with anaerobic PBS 2× under anaerobic conditions and diluted cells were inoculated into the assay tubes (10^6^ CFU ml^−1^ starting density). After 8 h, the invader strains were washed anaerobically as for the resident and inoculated in the assay tube (10^4^ CFU ml^−1^ starting density). The inocula were diluted and plated, and time points were taken and plated on the appropriate antibiotics (LB plus chloramphenicol or ampicillin) as for the aerobic assays, but samples were aspirated using needles and syringes to keep the assay tube anaerobic.

### Community invasion experiments (anaerobic)

As for anaerobic invasion experiments with only *E. coli* strains, buffered mGAM in Hungate tubes were used for community invasion experiments. Before community assembly, all symbiont strains in the 15-species community (Supplementary Table 1) were inoculated from glycerol stocks in mGAM anaerobically and allowed to grow to stationary phase (24–72 h depending on the symbiont). All symbiont strains were passaged (1:50) into fresh mGAM tubes 24 h before the start of the experiment. The resident strain was inoculated from a single colony and grown overnight.

As before, the resident strain was washed anaerobically in PBS 2× and seeded into the Hungate assay tubes containing 5 ml buffered mGAM at 10^6^ CFU ml^−1^ starting density. Subsequently, 100 µl of each desired symbiont was added to the assay tube using needles and syringes (fresh needle for each tube to avoid cross-contamination) and grown for 24 h at 37 °C and 220 rpm. Overnight cultures of the invader strains grown overnight from a single colony in mGAM were washed anaerobically with PBS and added to the assay tubes 24 h after the resident strain (10^4^ CFU ml^−1^ starting density). Selective plating was used to enumerate population sizes of the focal competing *E. coli* strains (resident strains on LB plus ampicillin, invader strains on LB plus chloramphenicol) as indicated in Fig. 4.

For nutrient supplementation experiments under anaerobic conditions (for example, Fig. 4g,h), the experimental set-up was the same, except the media used was ½ mGAM buffered with 50 mM MES to pH 6.2 with or without 1% sorbitol. 2.5 ml Hungate tubes with sterile pre-reduced buffered mGAM were prepared, and separately, sterile Milli-Q water and 2% sorbitol were prepared and reduced in the anaerobic chamber. Then, 2.5 ml of either Milli-Q water or 2% sorbitol was added to the mGAM tubes using a needle and syringe to create the desired media.

### Whole-genome sequencing

An overnight culture of *E. coli* 0960 and 0268 was prepared in LB containing 100 μg ml^−1^ ampicillin and 50 µg ml^−1^ kanamycin. A pellet was taken and DNA was extracted using ethanol precipitation and AMPure clean-up. Pellets were resuspended in nuclease-free water and transferred to a lysing matrix B tube (MP Biomedicals) and lysed using bead-beating (1 min at 25 Hz). Samples were centrifuged at high speed, and sodium acetate (1/10 volume) and ice-cold ethanol (96–100%; volume equal to the total amount of supernatant) were added to the supernatant; the mixture was incubated at −20 °C overnight. Samples were centrifuged at high speed, and the pellet was washed twice with 70% ethanol, before being dried and resuspended in nuclease-free water. Samples were then mixed with AMPure XP beads (Beckman Coulter) and incubated at room temperature for 5 min. Samples were washed twice with 70% ethanol using a magnet to avoid removing DNA bound to the AMPure beads. Following an air-drying step, nuclease-free water was added to the beads to resuspend DNA into the supernatant.

Source Biosciences performed whole-genome Illumina sequencing on a NovaSeq 600 to generate 10 million 150-bp paired-end reads. Genome assembly was performed with Unicycler v0.4.8 using default settings and subjected to protein family annotation^17^.

### Genomic analysis using protein family overlap

Genomic information from the eight *E. coli* strains was retrieved from the Bacterial and Viral Bioinformatics Resource Center (BV-BRC) database^61^ or, in the case of *E. coli* 0268 and *E. coli* 0960, we sequenced them (see above). Using methods we previously established^17^, we applied a cluster-based analysis grouping proteins encoded in genomes into BV-BRC global protein families^62^. For each pairwise interaction between an invader *E. coli* isolate and a resident AMR *E. coli* isolate, we computed the overlap between the two strains. Invader unique protein families indicate the protein families that are present in the invader strain that the resident strain does not have.

### Nutrient utilization using Biolog assays

All symbiont strains and the *E. coli* strains IAI1 and 0018 were previously analysed on Biolog AN MicroPlates (Biolog)^17^. Using the same method, we profiled the additional *E. coli* strains in this study (*E. coli* K12, Z1269, Z1331, HS, 0960, 0268). Briefly, overnight cultures in mGAM grown under anaerobic conditions were centrifuged and washed and diluted in AN inoculating fluid corresponding to 65% transmittance (OD 0.187) according to the manufacturer’s instructions. The inoculating fluid was seeded into the Biolog plates under aerobic conditions and then put in an airtight container with a GasPak EZ Container System Sachet to generate hydrogen-free anaerobic conditions. After 24 h at 35 °C without shaking, plates were measured at 590 nm. The absorbance reading at 590 nm (Abs 590 nm) was subtracted from the no carbon source control in well A1. Each strain was measured three times and the median blank-subtracted absorbance value was taken. As before^17^, we used a thresholding approach that defined a strain as being able to metabolize a given carbon source if the Abs 590 nm value was greater than 0.1 after blank subtraction (Extended Data Fig. 9a).

### Predictions based on Biolog assays

We computationally assembled all possible combinations of the 15 species (Supplementary Table 1) at distinct diversity levels (0, 1, 3, 5 and 15 species in addition to the resident AMR *E. coli* 0960 and the invader *E. coli* Z1269). Carbon source overlap was determined as follows: for each community, if any of the species within the community (the resident strain or any of the symbiont strains) used a given nutrient based on the Biolog measurements (Extended Data Fig. 9a) that the invader could also use, we considered there to be carbon source overlap. We did this procedure for all 95 carbon sources on the Biolog plate and reported the overlap as a percentage for each community (Fig. 4d).

We experimentally validated the predicted highest and lowest carbon source overlapping communities in Fig. 4e,f, choosing five communities at each diversity level of three and five species in addition to the focal *E. coli* (picking communities at random if there were ties in predicted carbon source overlap). There was one exception, which is that H3–5 is actually the community with the sixth highest overlap. This was used instead of one of the communities with higher overlap owing to technical reasons involving contamination during a pilot experiment in which the six best communities were screened for their ability to resist invasion. Supplementary Table 4 shows the identify of the species within these communities and the predicted carbon source overlap based on the Biolog plate measurements.

### Statistical analysis

All graphs and statistical analyses were performed using GraphPad Prism v10.2.2. Protein family overlap analyses and carbon source overlap predictions were computed in R v4.0.5 (ref. ^63^). The figure legends indicate the statistical tests used and the sample sizes. Non-parametric tests were generally used to avoid assumptions of normality.

### Reporting summary

Further information on research design is available in the Nature Portfolio Reporting Summary linked to this article.

## Supplementary information

Supplementary Information Supplementary text associated with the mathematical model and supplementary references.

Reporting Summary

Supplementary Tables 1–4 Bacterial strains, plasmids, primers and communities used in this study.

## Source data

Source Data Fig. 2 Raw data used to generate Fig. 2.

Source Data Fig. 3 Raw data used to generate Fig. 3.

Source Data Fig. 4 Raw data used to generate Fig. 4.

Source Data Extended Data Fig. 6 Raw data used to generate Extended Data Fig. 6.

Source Data Extended Data Fig. 7 Raw data used to generate Extended Data Fig. 7.

Source Data Extended Data Fig. 8 Raw data used to generate Extended Data Fig. 8.

Source Data Extended Data Fig. 9 Raw data used to generate Extended Data Fig. 9.

Source Data Extended Data Fig. 10 Raw data used to generate Extended Data Fig 10.

## Data Availability

All experimental data used to generate plots are available via Figshare at 10.6084/m9.figshare.29402243 (ref. ^64^). The whole-genome sequences of *E. coli* 0960 (NCBI accession SAMN49384365) and 0268 (NCBI accession SAMN49382617) have been deposited in Sequence Read Archive under BioProject PRJNA1279092. There are no restrictions on use of data, materials or code with the exception of *E. coli* strains 19Y000018, 18Y000960 and 19Y000268, which are protected by a material transfer agreement and requires permission from Nottingham University Hospitals Pathogen Bank. Source data are provided with this paper.

## Code availability

All code is available via GitHub at https://github.com/vit-pi/strain-replacement.
