# Supplementary material for: Strain displacement in microbiomes via ecological competition
Source: Nat Microbiol. 2025 Nov 7;10(12):3122–35. doi: 10.1038/s41564-025-02162-w (PMC12669036; doi:10.1038/s41564-025-02162-w)
Supplement: Supplementary file 1 — Supplementary text associated with the mathematical model and supplementary references. [file 41564_2025_2162_MOESM1_ESM.pdf]

nature microbiology

Supplementary information

https://doi.org/10.1038/s41564-025-02162-w

Strain displacement in microbiomes via ecological competition

In the format provided by the authors and unedited

Supplementary Text:

No invasion theorem

In this section, we prove the key mathematical result for ecological invasions into well-mixed and stable bacterial communities: **the invader cannot increase its invasion success by utilizing bacterial weapons if it invades from low density**. Intuitively, invasion success is governed solely by the initial growth rate of the invader. Crucially, bacterial weapons decrease the growth of the attacked community members but do not immediately improve the initial growth rate of the invader. On the contrary, if weapon production is costly, then the initial growth rate of the invader decreases with weapon production. Therefore, the invader cannot invade the community with a weapon unless it can invade it without a weapon.

To formalize this result, we consider consumer-resource models that are widely used to model well-mixed bacterial communities and their chemical environment[29,64]. We assume that bacteria can utilize different nutrient sources[17], exchange nutrients via cross-feeding[65] or participate in bacterial warfare by producing bacterial weapons[18], which can be broadly classified as contact-dependent weapons that require physical contact between bacterial cells, or diffusible weapons. The resulting population dynamics can be described by a general consumer-resource model as presented in the main text

$$\begin{aligned} \frac{dN_\sigma}{dt} &= N_\sigma(\lambda_\sigma(\mathbf{N},\mathbf{x}) - \delta_\sigma) \\ \frac{\mathrm{d}x_i}{dt} &= g_i(\mathbf{N},\mathbf{x}) - \textstyle\sum_\sigma N_\sigma\, d_{i\sigma}(\mathbf{N},\mathbf{x}), \end{aligned} \qquad (1)$$

where N_σ is the abundance of strain σ and x_i the concentration of a chemical species i (nutrient or toxin). Defining the vectors of strain abundance $\mathbf{N} = (N_1, \dots)$ and chemical concentration $\mathbf{x} = (x_1, \dots)$, $\lambda_\sigma(\mathbf{N},\mathbf{x})$ denotes the bacterial growth rate, δ_σ the bacterial dilution rate, $g_i(\mathbf{N},\mathbf{x})$ the net chemical import rate and $d_{i\sigma}(\mathbf{N},\mathbf{x})$ the net uptake rate of chemical i by strain σ. With appropriate choice of the rate functions, this model can capture

- nutrient utilization: the growth rate λ_σ of strain σ has a positive dependence on nutrient concentrations x_i,
- cross-feeding: the production rate g_i of nutrient i has a positive dependence on the abundance N_σ of the nutrient-producing strain σ,
- contact weapons: the growth rate λ_σ of the attacked strain σ has a negative dependence on the abundance N_τ of the attacking strain τ,

- diffusible weapons: the import rate g_i of toxin i has a positive dependence on the abundance N_τ of the toxin-producing strain τ, while the growth rate λ_σ of the toxin-affected strain σ has a negative dependence on the concentration x_i of the toxin i.

Importantly, the consumer-resource model in **Eq. 1** can be utilized to study ecological invasions. In particular, we consider a community of strains $\sigma \in \Sigma$, invader strain $\tau \notin \Sigma$ and any chemical species $i \in I$ that any of these strains exchange with their environment. We assume that the system in **Eq. 1** describes the dynamics of community strains Σ and chemicals I, which are assumed to have reached a stable ecological equilibrium $\mathbf{N}'$, $\mathbf{x}'$. Furthermore, we extend the dynamical system in **Eq. 1** to include the invader strain τ with abundance N_τ. The dynamics of the extended system with strains $\Sigma \cup \{\tau\}$ is described by equations of the same form as **Eq. 1**, but with extended rate functions $\lambda_\sigma^+(N_\tau, \mathbf{N}, \mathbf{x})$, $g_i^+(N_\tau, \mathbf{N}, \mathbf{x})$ and $d_{i\sigma}^+(N_\tau, \mathbf{N}, \mathbf{x})$ for $\sigma \in \Sigma \cup \{\tau\}$ and $i \in I$ that satisfy

$$\begin{aligned} \lambda_\sigma^+(0, \mathbf{N}, \mathbf{x}) &= \lambda_\sigma(\mathbf{N}, \mathbf{x}), \\ g_i^+(0, \mathbf{N}, \mathbf{x}) &= g_i(\mathbf{N}, \mathbf{x}), \\ d_{i\sigma}^+(0, \mathbf{N}, \mathbf{x}) &= d_{i\sigma}(\mathbf{N}, \mathbf{x}), \end{aligned} \qquad \text{(S1)}$$

for any community strain $\sigma \in \Sigma$ and any chemical species $i \in I$. With these definitions, we can formulate the main result.

Theorem 1 (No invasion): *The strain $\tau \notin \Sigma$ can invade the ecological equilibrium $\boldsymbol{N}', \boldsymbol{x}'$ of the community Σ from low density if and only if*

$$\lambda_\tau^+(0, \boldsymbol{N}', \boldsymbol{x}') > \delta_\tau, \qquad \text{(S2)}$$

which, up to the vector notation, is the same as equation (2) in the main text. Consequently, the invader strain τ that is equipped with a weapon cannot invade the community Σ unless it can invade it without the weapon. Moreover, its invasibility depends: (a) negatively on the abundance of resident strains that carry a contact-dependent weapon against the invader, (b) negatively on the equilibrium abundance of toxins left in medium to which the invader is susceptible, and (c) positively on the equilibrium abundance of nutrients left in the medium which the invader can utilize.

Proof: Due to the relationships in **Eq. S1**, the point $N_\tau = 0$, $\mathbf{N} = \mathbf{N}'$ and $\mathbf{x} = \mathbf{x}'$ is a fixed point of the extended dynamical system with strains $\Sigma \cup \{\tau\}$. The stability of this fixed point determines the invasibility of strains τ from low density. Moreover, the relationships in **Eq. S1** imply that

$$
\begin{aligned}
\frac{\partial\lambda_\sigma^+(0,\boldsymbol{N}',\boldsymbol{x}')}{\partial N_\omega} &= \frac{\partial\lambda_\sigma(\boldsymbol{N}',\boldsymbol{x}')}{\partial N_\omega},\\
\frac{\partial\lambda_\sigma^+(0,\boldsymbol{N}',\boldsymbol{x}')}{\partial x_i} &= \frac{\partial\lambda_\sigma(\boldsymbol{N}',\boldsymbol{x}')}{\partial x_i},\\
\frac{\partial g_i^+(0,\mathbf{N}',\mathbf{x}')}{\partial N_\omega} &= \frac{\partial g_i(\boldsymbol{N}',\boldsymbol{x}')}{\partial N_\omega},\\
\frac{\partial g_i^+(0,\boldsymbol{N}',\boldsymbol{x}')}{\partial x_j} &= \frac{\partial g_i(\boldsymbol{N}',\boldsymbol{x}')}{\partial x_j},\\
\frac{\partial d_i^+(0,\boldsymbol{N}',\boldsymbol{x}')}{\partial N_\omega} &= \frac{\partial d_i(\boldsymbol{N}',\boldsymbol{x}')}{\partial N_\omega},\\
\frac{\partial d_i^+(0,\boldsymbol{N}',\boldsymbol{x}')}{\partial x_j} &= \frac{\partial d_i(\boldsymbol{N}',\boldsymbol{x}')}{\partial x_j},
\end{aligned}
\qquad \text{(S3)}
$$

for any $\sigma, \omega \in \Sigma$ and $i, j \in I$. Consequently, the Jacobian of the extended dynamical system at the fixed point $N_\tau = 0$, $\mathbf{N} = \mathbf{N}'$ and $\mathbf{x} = \mathbf{x}'$ is given by

$$J^+ = \begin{pmatrix} \lambda_\tau^+(0,\mathbf{N}',\mathbf{x}') - \delta_\tau & 0 \\ * & J \end{pmatrix}, \qquad \text{(S4)}$$

where J is the Jacobian of the reduced dynamical system for the community strains Σ and chemicals I evaluated at the fixed point $\mathbf{N}'$ and $\mathbf{x}'$. Since the matrix J^+ in equation (S5) is upper triangular, the characteristic polynomial for J^+ factorises,

$$\det(J^+ - zI) = (\lambda_\tau^+(0,\mathbf{N}',\mathbf{x}') - \delta_\tau - z)\det(J - zI), \qquad \text{(S5)}$$

and the eigenvalues of J^+ are given by $\lambda_\tau^+(0,\mathbf{N}',\mathbf{x}') - \delta_\tau$ and the union of eigenvalues of J. Since the community Σ is stable, the Jacobian J has eigenvalues with negative real parts. Therefore, the fixed point $N_\tau = 0$, $\mathbf{N} = \mathbf{N}'$ and $\mathbf{x} = \mathbf{x}'$ is unstable (i.e., the strain τ invades the community Σ from low density) if and only if **Eq. S2** holds.

Crucially, weapons produced by strain τ do not increase its growth rate λ_τ^+ but rather decrease the growth rate λ_σ^+ of susceptible competitor strains σ, either directly via N_τ-dependence for contact weapons or indirectly via x_i-dependence for diffusible weapons mediated by toxin i. Consequently, if $\tilde{\lambda}_\tau^+$ (resp. λ_τ^+) corresponds to the growth rate of the invader τ equipped with a weapon (resp. lacking a weapon), then

$$\tilde{\lambda}_\tau^+(0,\mathbf{N}',\mathbf{x}') \le \lambda_\tau^+(0,\mathbf{N}',\mathbf{x}'), \qquad \text{(S6)}$$

with equality if and only if there are no fitness costs associated with carrying the weapon. If the invader that lacks a weapon cannot invade the community, then **Eq. S2** implies that

$$\lambda_\tau^+(0,\mathbf{N}',\mathbf{x}') < \delta_\tau. \qquad \text{(S7)}$$

Therefore,

$$\tilde{\lambda}_\tau^+(0,\mathbf{N}',\mathbf{x}') \leq \lambda_\tau^+(0,\mathbf{N}',\mathbf{x}') < \delta_\tau \qquad \text{(S8)}$$

and **Eq. S2** implies that the invader τ that is equipped with a weapon cannot invade the community Σ. Furthermore, as the initial growth rate $\lambda_\tau^+(0,\mathbf{N}',\mathbf{x}')$ of the invader strain τ has a negative dependence on the abundance N_σ of any strain σ that carries a contact-dependent weapon against the invader, the abundance of such strains decreases the invasibility of the invader strain τ according to the invasion criterion in **Eq. S2**. Similarly, the initial growth rate $\lambda_\tau^+(0,\mathbf{N}',\mathbf{x}')$ of the invader strain τ has a negative (resp. positive) dependence on the abundance x_i of any toxin (resp. nutrient) i that is left over in the medium of the resident community and is potent against (resp. utilized by) the invader strain τ, implying that the abundance of such toxins (resp. nutrients) decreases (resp. increases) the invasibility of the invader strain τ according to the invasion criterion in **Eq. S2**. □

In summary, during the initial phase of invasion from low density, bacterial weapons do not provide an advantage to the invader strain in well-mixed cultures. This is true regardless of how toxin production functions $g_i(\mathbf{N},\mathbf{x})$ are chosen, therefore will remain true irrespective of any weapon regulation. Instead, the invasion success is driven by nutrient competition and the access of the invader to private nutrients, as explained in[17], as well as a lack of potent antimicrobials produced by the resident community against the invader strain. In the following section, we will illustrate these general principles for the case of a single resident strain and an invader strain that uses a single diffusible weapon.

Invasion into a single resident strain

In the previous section, we proved the key new result that characterizes ecological invasions into bacterial communities, see **Theorem 1**. In this section, we illustrate this result by studying a single resident strain that is being invaded by a potential producer of a single diffusible toxin that targets competing strains.

Equilibrium of the resident strain

Having introduced the model (**Methods, Eq. 3**), we can explore the ecology of the resident strain in the absence of the invader strain. If the invader is absent ($N_I = 0$), the toxins are absent ($y = 0$), the private nutrient of the invader reaches a steady state ($x_I = m_I/D$) and the dynamics of the resident N_R follows the equations

$$\begin{aligned} \dot{N}_R &= N_R\left(\frac{r_R x/k_R + R_R x_R/K_R}{1 + x/k_R + x_R/K_R} - \delta\right) \\ \dot{x} &= m - Dx - N_R\frac{c_R x/k_R}{1 + x/k_R + x_R/K_R} \\ \dot{x}_R &= m_R - Dx_R - N_R\frac{C_R x/K_R}{1 + x/k_R + x_R/K_R} \end{aligned} \qquad \text{(S9)}$$

Tilman's graphical approach[28,29] can be used to show that this system admits a unique stable equilibrium, provided that the dilution rate is smaller than the maximal possible growth rate

$$\frac{r_R m/k_R + R_R m_R/K_R}{D + m/k_R + m_R/K_R} > \delta. \quad \text{(S10)}$$

If we further assume that the dilution rate is small ($D \to 0$), then the unique stable equilibrium of the resident phenotype can be found analytically as

$$\begin{aligned} N_R &\approx \frac{1}{\delta}\left(\frac{r_R m}{c_R} + \frac{R_R m_R}{C_R}\right) \equiv N', \\ x &\approx k_R \frac{m\delta C_R}{(r_R-\delta)C_R m + (R_R-\delta)c_R m_R} \equiv x', \quad \text{(S11)} \\ x_R &\approx K_R \frac{m_R \delta c_R}{(r_R-\delta)C_R m + (R_R-\delta)c_R m_R} \equiv x_R'. \end{aligned}$$

Invasibility criterion

Having found the ecological equilibrium of the resident strain, we can use **Theorem 1** to formulate a mathematical criterion for the ecological invasion. In particular, the invader strain successfully invades from low density precisely when

$$\lambda_I^+(z) = (1-z)\frac{r_I x'/k_I + R_I m_I/DK_I}{1 + x'/k_I + m_I/DK_I} > \delta, \quad \text{(S12)}$$

where the initial growth rate of invaders $\lambda_I^+(z)$ corresponds to the growth rate of the invader with toxin investment z in the spent medium of the resident strain. Crucially, the invader growth rate $\lambda_I^+(z)$ decreases linearly with toxin investment z (**Fig. ED2A**) and is independent of toxin potency p (**Fig. ED2B**). Therefore, consistently with **Theorem 1**, the invasion is successful only if it is successful without investing into the weapon, that is

$$\lambda_I^+(0) > \delta. \qquad \text{(S13)}$$

This condition can be interpreted as a condition for metabolic diversity, determined by the growth $\lambda_I^+(0)$ of the invader strain that lacks a weapon on the spent medium of the resident strain. In particular, if the invader cannot grow on the spent medium due to a large metabolic overlap ($r_I x'/(k_I + x') < \delta$) and the invader does not have any available private nutrients ($m_I = 0$), then $\lambda_I^+(0) < \delta$ and the invader is diluted faster than it grows. In contrast, if the invader has a large amount of available private nutrients ($m_I \to \infty$) that can support its growth in the absence of the shared nutrients ($R_I > \delta$), then $\lambda_I^+(0) \approx R_I > \delta$ and the invader growth overcomes the dilution. Therefore, supplementation m_I of private nutrients to the invader promotes the success of invasion (**Fig. ED2A-B**).

In summary, there are two dynamically distinct cases

- metabolic overlap: Dilution overcomes growth of the invader in the spent medium of the resident, $\delta > \lambda_I^+(0)$, and the invader cannot invade the resident, irrespective of the investment z into toxin production.
- metabolic diversity: The growth of the invader in the spent medium of the resident overcomes dilution, $\delta < \lambda_I^+(0)$, and the invader can invade the resident provided the toxin investment does not impose a significant cost on the metabolism

$$0 \le z < 1 - \frac{\delta}{\lambda_I^+(0)} \equiv z_{\text{invade}}. \quad \text{(S14)}$$

Population dynamics after invasion

Once the invader successfully invades, it can either co-exist with the resident or displace it. Since the dynamics after the invasion phase is not analytically tractable, the possibility of coexistence must be studied numerically, for example by a numerical search for a positive fixed point in **Eq. 3**. Despite this limitation, if each strain can invade the resident population of the other strain, both strains must co-exist. Therefore, the mutual invasibility of strains provides a sufficient condition for co-existence that can be studied analytically. Moreover, numerics suggest that mutual invasibility is also necessary for co-existence if private nutrients are abundant (**Fig. 1; Fig. ED1**).

To study mutual invasibility, we can reverse the roles of the invader and resident in the previous calculations and notice that the dynamics of the original invader in the absence of the original resident ($N_R = 0, x_R = m_R/D$) is given by

$$\begin{aligned} \dot{N}_I &= N_I\left((1-z)\frac{r_I x/k_I + R_I x_I/K_I}{1 + x/k_I + x_I/K_I} - \delta\right) \\ \dot{x} &= m - Dx - N_I(1-z)\frac{c_I x/k_I}{1 + x/k_I + x_I/K_I} \\ \dot{x}_I &= m_I - Dx_I - N_I(1-z)\frac{C_I x_I/K_I}{1 + x/k_I + x_I/K_I} \\ \dot{y} &= zgN_I - dy. \end{aligned} \quad \text{(S15)}$$

This system admits a unique stable equilibrium that can be found analytically, provided dilution rates are small ($D, d \to 0$)

$$\begin{aligned} N_I &\approx \frac{1}{\delta}\left(\frac{r_I m}{c_I} + \frac{R_I m_I}{C_I}\right) \equiv N'', \\ x &\approx k_I \frac{m\delta C_I}{(r_I(1-z)-\delta)C_I m + (R_I(1-z)-\delta)c_I m_I} \equiv x'', \\ x_I &\approx K_I \frac{m_I \delta c_I}{(r_I(1-z)-\delta)C_I m + (R_I(1-z)-\delta)c_I m_I} \equiv x_I''. \\ y &\approx \frac{zg}{d\delta}\left(\frac{r_I m}{c_I} + \frac{R_I m_I}{C_I}\right) \equiv y''. \end{aligned} \quad \text{(S16)}$$

Using **Theorem 1**, we can deduce that the original resident can invade the original invader precisely if

$$\lambda_R^{+}(z,p) = \frac{r_R x''/k_R + R_R m_R/DK_R}{1 + x''/k_R + m_R/DK_R} - \frac{p y''}{y'' + K} > \delta. \qquad \text{(S17)}$$

The growth $\lambda_R^{+}(z,p)$ can be interpreted as the growth of the resident in the spent medium of the invader that produced toxins of potency p after investment at level z.

Since mutual invasibility implies co-existence, the resident strain can be displaced by a successful invader only if the condition in **Eq. S17** is violated. Crucially, the resident growth $\lambda_R^{+}(z,p)$ is a generally non-monotonic function of toxin investment z (**Fig. ED2C**) and a linearly decreasing function of toxin potency p (**Fig. ED2D**). While small levels of toxin investment z reduce the growth of the resident strain, large levels are costly for the metabolism of the invader strain, improving the competitive advantage of the resident strain. Consequently, if the invader strain invests suitably in producing a sufficiently potent toxin, then there is an increased chance of displacing the resident strain after a successful initial invasion. Furthermore, increased supplementation of private nutrients to the invader strain can promote the displacement of the resident strain (**Fig. ED2C-D**).

Summary

In summary, we identified the condition when the invader strain can successfully invade a population of a resident strain from low density (section "**Invasibility criterion**"), as well as a necessary condition for the subsequent displacement of the resident strain (section "**Population dynamics after invasion**"). These conditions are summarized in **Fig. ED2** and can be used to construct phase diagrams that relate metabolic diversity and toxin production to the outcome of the invasion dynamics (**Fig. 1B, Fig. ED1A,F,K**). In conclusion, the success of invasion depends critically on the availability of private nutrients and is independent of toxin investment or potency, consistently with the general result in **Theorem 1** (**Fig. 1, Fig. ED1**). Moreover, a very large investment into toxin production can slow down the metabolism and impair the success of invasion. After a successful initial invasion, the invader can displace the resident if it produces a sufficiently potent toxin at a suitable level. Importantly, further supplementation of private nutrients to the invader can be critical, as a sufficient supplementation of private nutrients is necessary to empower the production of toxins (**Fig. 1B, Fig. ED1**). We also note that the supplementation of a private nutrients is not necessary if the invaders come with density larger than that of the residents and invest sufficiently in weapons to displace them (**Fig. ED4**). This observation suggests that the major benefit of supplementing the invaders with a private nutrient lies in boosting their initially small abundances, thereby allowing the invaders' weapons to become effective.

Spatial model

No invasion theorem

In this section, we prove that **Theorem 1** extends to spatial contexts where bacteria, nutrients and toxins diffuse through space. In particular **Eq. 1** can be extended to spatial contexts by replacing the

total abundances of bacterial strains $N_\sigma(t)$ and chemical species $x_i(t)$ by their spatial densities $N_\sigma(t, \boldsymbol{z})$ and $x_i(t, \boldsymbol{z})$, where $\boldsymbol{z} \in \mathbb{R}^D$ describes a spatial position on a line ($D = 1$), plane ($D = 2$), or in a three-dimensional space ($D = 3$). For simplicity, we assume that the spatial domain is infinitely large, but we note the following results can be easily generalized to finite domains with appropriate boundary conditions (reflective or periodic)[66]. With these changes, **Eq. 1** becomes

$$\begin{aligned} \frac{\partial N_\sigma}{\partial t} &= M_\sigma \nabla^2 N_\sigma + N_\sigma(\lambda_\sigma(\mathbf{N}, \mathbf{x}) - \delta_\sigma) \\ \frac{\partial x_i}{\partial t} &= M_i \nabla^2 x_i + g_i(\mathbf{N}, \mathbf{x}) - \textstyle\sum_\sigma N_\sigma\, d_{i\sigma}(\mathbf{N}, \mathbf{x}), \end{aligned} \qquad \text{(S18)}$$

where M_σ is the diffusivity of a bacterial strain σ, M_i is the diffusivity of a chemical species i, and $\nabla^2 = \partial_{z_1}^2 + \cdots + \partial_{z_D}^2$ is the D-dimensional Laplacian operator. To study the invasion of a new strain, we consider a community of strains $\sigma \in \Sigma$, invader strain $\tau \notin \Sigma$ and all chemical species $i \in I$ that any of these strains exchange with their environment. With these definitions, Theorem 1 can be extended to spatial contexts as follows:

Theorem 2 (Spatial No invasion): *Assume that the ecological equilibrium of the resident community is stable and spatially homogeneous, that is $N_\sigma(t, \boldsymbol{z}) = N_\sigma{}'$ and $x_i(t, \boldsymbol{z}) = x_i{}'$ for some constants $N_\sigma', x_i{}'$. Then, the strain $\tau \notin \Sigma$ can invade the ecological equilibrium of the community Σ from low density in the spatial system given by **Eq. S18** precisely if **Eq. 2** in **Theorem 1** is satisfied, that is precisely if it can invade the well-mixed system given by equation **Eq. 1**.*

Proof: Consider small perturbations $\epsilon_\sigma(\boldsymbol{z})$, $\epsilon_i(\boldsymbol{z})$ and $\epsilon_\tau(\boldsymbol{z})$ around the spatially homogeneous equilibrium $N_\sigma(t, \boldsymbol{z}) = N_\sigma{}'$, $x_i(t, \boldsymbol{z}) = x_i{}'$ and $N_\tau(t, \boldsymbol{z}) = 0$ given by $N_\sigma(t, \boldsymbol{z}) = N_\sigma' + \epsilon_\sigma(\boldsymbol{z})$, $x_i(t, \boldsymbol{z}) = x_i' + \epsilon_i(\boldsymbol{z})$ and $N_\tau(t, \boldsymbol{z}) = \epsilon_\tau(\boldsymbol{z})$. Linearization of the system in **Eq. S18** yields

$$\frac{\partial}{\partial t}\begin{pmatrix} \epsilon_\tau \\ \epsilon_\sigma \\ \epsilon_i \end{pmatrix} = M^+ \nabla^2 \begin{pmatrix} \epsilon_\tau \\ \epsilon_\sigma \\ \epsilon_i \end{pmatrix} + J^+ \begin{pmatrix} \epsilon_\tau \\ \epsilon_\sigma \\ \epsilon_i \end{pmatrix}, \quad \text{(S19)}$$

where $M^+ = diag(M_\tau, M_\sigma, M_i)$ is a matrix with diffusivities on the diagonal and J^+ is the Jacobian matrix of the well-mixed system as given by **Eq. S4**. Upon Fourier transformation

$$\begin{pmatrix} \epsilon_\tau(\boldsymbol{k}) \\ \epsilon_\sigma(\boldsymbol{k}) \\ \epsilon_i(\boldsymbol{k}) \end{pmatrix} = \int \begin{pmatrix} \epsilon_\tau(\boldsymbol{z}) \\ \epsilon_\sigma(\boldsymbol{z}) \\ \epsilon_i(\boldsymbol{z}) \end{pmatrix} e^{-i\boldsymbol{kz}}\, d\boldsymbol{z}, \quad \text{(S20)}$$

the system in **Eq. S19** becomes

$$\frac{d}{dt}\begin{pmatrix} \epsilon_\tau(\boldsymbol{k}) \\ \epsilon_\sigma(\boldsymbol{k}) \\ \epsilon_i(\boldsymbol{k}) \end{pmatrix} = (-k^2 M^+ + J^+) \begin{pmatrix} \epsilon_\tau(\boldsymbol{k}) \\ \epsilon_\sigma(\boldsymbol{k}) \\ \epsilon_i(\boldsymbol{k}) \end{pmatrix}, \quad \text{(S21)}$$

where $\boldsymbol{k} = (k_1, \dots, k_D)$ is a vector of wavenumbers for each spatial dimension and $k^2 = \boldsymbol{k} \cdot \boldsymbol{k}$ is its squared length. Therefore, the invader can invade from low density precisely if the spatially extended Jacobian matrix

$$J(k^2) = -k^2 M^+ + J^+, \text{ (S22)}$$

has a positive eigenvalue for some wavenumber k^2. Defining $M = diag(M_\sigma, M_i)$ to be the diffusivity matrix without the invader strain, **Eq. 4** implies that

$$J(k^2) = -k^2 M^+ + J^+ = \begin{pmatrix} \lambda_\tau^+(0, \mathrm{N}'_\sigma, x'_\sigma) - \delta_\tau - M_\tau k^2 & 0 \\ * & J - Mk^2 \end{pmatrix}, \qquad \text{(S23)}$$

where J is the Jacobian of the reduced dynamical system for the community strains Σ and chemicals I evaluated at the stable and spatially homogeneous steady state. Since the matrix $J(k^2)$ in equation (S23) is upper triangular, its characteristic polynomial factorizes into

$$\det(J^+ - zI) = (\lambda_\tau^+(0, \mathrm{N}'_\sigma, x'_\sigma) - \delta_\tau - M_\tau k^2 - z)\det(J - Mk^2 - zI), \qquad \text{(S24)}$$

implying that the eigenvalues of $J(k^2)$ are given by $\lambda_\tau^+(0, \mathrm{N}'_\sigma, x'_\sigma) - \delta_\tau - M_\tau k^2$ and the eigenvalues of $J - Mk^2$. Since the spatially homogeneous steady state is stable, the Jacobian $J - Mk^2$ has eigenvalues with negative real parts. Therefore, the invaders can invade from low density precisely if

$$A(k^2) = \lambda_\tau^+(0, \mathrm{N}'_\sigma, x'_\sigma) - \delta_\tau - M_\tau k^2 > 0, \quad \text{(S25)}$$

for some wavenumber k^2. Since $A(k^2)$ decreases linearly with $k^2 \geq 0$, this condition is satisfied precisely when

$$A(0) = \lambda_\tau^+(0, \mathrm{N}'_\sigma, x'_\sigma) - \delta_\tau 0 > 0, \quad \text{(S26)}$$

which is equivalent to **Eq. 2**. □

Invasion into a single resident strain

In this section, we will illustrate the general spatial theory in **Eq. S18** in the specific context of invasion into a single resident strain. In particular, the dynamics of **Eq. 3** generalizes into spatially explicit contexts as

$$
\begin{aligned}
\frac{\partial \mathrm{N_R}}{\partial \mathrm{t}} &= M_{NR}\nabla^2 N_R + N_R\left(\frac{r_R x/k_R + R_R x_R/K_R}{1 + x/k_R + x_R/K_R} - \frac{py}{y+K} - \delta\right) \\
\frac{\partial \mathrm{N_R}}{\partial \mathrm{t}} &= M_{NI}\nabla^2 N_I + N_I\left((1-z)\frac{r_I x/k_I + R_I x_I/K_I}{1 + x/k_I + x_I/K_I} - \delta\right) \\
\frac{\partial \mathrm{x}}{\partial \mathrm{t}} &= M_x\nabla^2 x + m - Dx - N_R\frac{c_R x/k_R}{1 + x/k_R + x_R/K_R} - N_I(1-z)\frac{c_I x/k_I}{1 + x/k_I + x_I/K_I} \\
\frac{\partial \mathrm{x_R}}{\partial \mathrm{t}} &= M_{xR}\nabla^2 x_R + m_R - Dx_R - N_R\frac{C_R x/K_R}{1 + x/k_R + x_R/K_R} \\
\frac{\partial \mathrm{x_I}}{\partial \mathrm{t}} &= M_{xI}\nabla^2 x_I + m_I - Dx_I - N_I(1-z)\frac{C_I x_I/K_I}{1 + x/k_I + x_I/K_I} \\
\frac{\partial \mathrm{y}}{\partial \mathrm{t}} &= M_y\nabla^2 y + zgN_I - dy - N_R\frac{sy}{y+K}.
\end{aligned}
\qquad \text{(S27)}
$$

To illustrate the key concepts, we consider a 1-dimensional space of length $L = 1$ with reflective boundary conditions and simulate **Eq. S27** numerically by the method of lines. As suggested by **Theorem 2,** the resulting invasion dynamics is very similar to the well-mixed contexts (compare **Fig. 1** to **Fig. ED5**). Specifically, invasion is only successful if the invading strain has access to private nutrients. Moreover, the successful invasion is followed by the displacement of the resident strain precisely if the invading strain produces a toxin in addition to having the access to its private nutrient. The spatially explicit model extends the results of the well-mixed model by showcasing the spatial profiles of invaders and residents in space over time. In particular, it can be noticed that successful invaders form a travelling wave that slowly fills the space. Moreover, in cases where the invaders displace the residents, the extending wave of invaders is balanced by a receding wave of residents with a clear interface between the two strains, until the residents are pushed away from the space completely.

Supplementary references

64 Marsland, R., Cui, W., Goldford, J. & Mehta, P. The Community Simulator: A Python package for microbial ecology. *PLoS One* **15**, e0230430 (2020). https://doi.org/10.1371/journal.pone.0230430

65 D'Souza, G. *et al.* Ecology and evolution of metabolic cross-feeding interactions in bacteria. *Nat Prod Rep* **35**, 455-488 (2018). https://doi.org/10.1039/c8np00009c

66 Klika, V., Kozák, M. & Gaffney, E. A. Domain Size Driven Instability: Self-Organization in Systems with Advection. *SIAM Journal on Applied Mathematics* **78**, 2298-2322 (2018). https://doi.org/10.1137/17m1138571

67 Pugsley, A. P. & Schwartz, M. A genetic approach to the study of mitomycin-induced lysis of Escherichia coli K-12 strains which produce colicin E2. *Mol Gen Genet* **190**, 366-372 (1983). https://doi.org/10.1007/BF00331060

68 Harms, A. *et al.* A bacterial toxin-antitoxin module is the origin of inter-bacterial and inter-kingdom effectors of Bartonella. *PLoS genetics* **13**, e1007077 (2017).

69 Formal, S. B., Dammin, G. J., Labrec, E. H. & Schneider, H. Experimental Shigella infections: characteristics of a fatal infection produced in guinea pigs. *J Bacteriol* **75**, 604-610 (1958). https://doi.org/10.1128/jb.75.5.604-610.1958

70 Wotzka, S. Y. *et al.* Microbiota stability in healthy individuals after single-dose lactulose challenge-A randomized controlled study. *PLoS One* **13**, e0206214 (2018). https://doi.org/10.1371/journal.pone.0206214

71 Benz, F. *et al.* Plasmid- and strain-specific factors drive variation in ESBL-plasmid spread in vitro and in vivo. *ISME J* (2020). https://doi.org/10.1038/s41396-020-00819-4
